# Efficient Polaron Recombination and Fast Energy Transfer in a Deep Blue Phosphorescent Pt(II) Complex via Covalently Fused p‐Type Host

**DOI:** 10.1002/advs.76331

**Published:** 2026-06-28

**Authors:** You Na Song, Bubae Park, Garam Han, Eun Bi Kim, Junseop Lim, Wan Pyo Hong, Sunwoo Kang, Jae‐Min Kim, Hyung Youn Oh, Taekyung Kim

**Affiliations:** ^1^ Department of Chemical Engineering Kyung Hee University Yongin Gyeonggi Republic of Korea; ^2^ LORDIN Hwaseong Gyeonggi Republic of Korea; ^3^ Humboldt Centre for Nano‐ and Biophotonics Institute For Light and Matter Department of Chemistry University of Cologne Köln Germany; ^4^ Department of Chemistry Gachon University Seongnam Gyeonggi Republic of Korea; ^5^ Department of Chemistry Dankook University Cheonan Chungnam Republic of Korea; ^6^ Department of Advanced Materials Engineering Chung‐Ang University Anseong Gyeonggi Republic of Korea

**Keywords:** blue phosphorescence, fused emitter, organic light‐emitting diodes, platinum complex, zero‐radius of intramolecular energy transfer mechanism

## Abstract

Achieving high‐efficiency deep‐blue phosphorescent organic light‐emitting diodes is fundamentally hindered by intermolecular kinetic bottlenecks in polaron recombination and exciton‐transfer dynamics. We overcome these limits through a fused emitter architecture where a p‐type host and a tetradentate Pt(II) dopant are covalently integrated into a single molecule, Pt‐SP‐tCz. Despite direct chemical integration, two units retain fully independent photophysical and electrochemical identities, establishing dual recombination sites within a single molecule, significantly enhancing radiative probability. The fused geometry collapses energy donor–acceptor separation to the molecular length scale, enabling exceptionally fast intramolecular energy‐transfer pathway—zero‐radius intramolecular energy transfer (ZRIET). When paired with an n‐type host, the intramolecular p‐type host facilitates exciplex formation, creating a second zero‐radius pathway that transfers excitons directly to the Pt center—namely, the ZETPLEX mechanism. This dual short‐range transfer framework produces a record‐high Förster resonance energy transfer rate of 3.64 × 10^8^ s^−^^1^, and a recombination coefficient of 1.12 × 10^−7^ cm^3^ s^−1^ unattainable in conventional exciplex systems. A simplified two‐component EML achieves an external quantum efficiency of 23.6% with deeper‐blue emission and stability comparable to the optimized three‐component devices. Our results demonstrate that molecular fusion provides a powerful strategy to simultaneously transcend intermolecular kinetic limits, establishing a new paradigm for high‐performance deep‐blue emission.

## Graphical abstract

Achieving high‐efficiency deep‐blue phosphorescent OLEDs is limited by intermolecular kinetic bottlenecks. We overcome this using a single‐molecule fused emitter architecture, Pt‐SP‐tCz, combining a p‐type host and Pt(II) dopant. This design enables zero‐radius intramolecular energy transfer (ZRIET) and a novel ZETPLEX mechanism. Consequently, it delivers record‐high transfer rates and an external quantum efficiency of 23.6% in simplified two‐component devices.

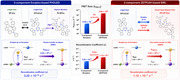

## 1 Introduction

While organic light‐emitting diodes (OLEDs) have firmly established themselves as a cornerstone technology in the display industry, the development of high‐efficiency blue OLEDs remains a critical and ongoing challenge [1, 2, 3]. The development of blue phosphorescent OLEDs (PhOLEDs) employing phosphorescent emitters based on iridium (Ir) or platinum (Pt) complexes has opened a pathway to high‐efficiency blue OLEDs by utilizing the heavy atom effect to harness triplet excitons and theoretically achieve an internal quantum efficiency of up to 100% [4, 5]. However, the development of high‐efficiency deep‐blue phosphorescent OLEDs continues to be impeded by two fundamental limitations rooted in the *intermolecular* nature of the emitting layer (EML): (i) slow and inefficient polaron recombination, and (ii) substantial energy losses during exciton transfer from the host to the dopant [6, 7, 8]. In conventional architectures, a p‐type host, an n‐type host, and a phosphorescent dopant must operate cooperatively to facilitate charge injection, exciton generation, and exciton transfer. Each of these processes is constrained by the spatial separation between molecules, which restricts electronic coupling, suppresses polaron recombination [9, 10, 11], and introduces substantial losses during exciton transfer [4, 12]. These limitations are particularly detrimental in the deep‐blue region, where the high exciton energies exacerbate non‐radiative decay and accelerate chemical degradation. Thus, the bottleneck in blue PhOLEDs is not simply the design of more efficient emitters, but the intrinsic requirement that exciton management occurs across multiple independent molecules separated by distances that fundamentally limit the rates at which energy can flow. Recent advances in mixed‐host and sensitized architectures have partially alleviated exciton‐management issues [13, 14], yet the inherent intermolecular nature of these processes imposes unavoidable spatial and energetic losses. Fundamentally, new concepts are required to escape the limitations imposed by molecular separation.

A novel fused emitter, Platinum(II)[6‐(1,3‐Dihydro‐3‐(3,5‐di‐tert‐butylphenyl)‐2H‐imidazol‐2‐ylidene‐κC^2^)‐1,2‐phenylene‐κC [1–3]]‐oxy[10'‐(tert‐butyl)‐5‐(4‐(tert‐butyl)pyridin‐2‐yl‐κN)‐5'‐phenyl‐5H,5'H‐12,12'‐spirobi[indeno[1,2‐c]carbazole‐6,7‐diyl‐κC [1–3]] (Pt‐SP‐tCz), was successfully designed and synthesized by integrating a p‐type host with a tetradentate Pt(II) dopant. Comprehensive optical and electrical characterizations confirm that the p‐type host unit and tetradentate Pt(II) dopant unit in Pt‐SP‐tCz are structurally fused yet retain their individual photophysical and electrochemical functionalities without forming new hybridized characteristics. This electronic decoupling yields two independent oxidation channels and consequently dual recombination sites—a feature not available in conventional dopants, which typically act as hole traps. Molecular fusion forces the donor and acceptor into the same molecular framework, collapsing their separation to the molecular scale and thereby enabling an ultrafast *zero‐radius intramolecular energy transfer* (ZRIET) route [15, 16].

Even more importantly, the integrated p‐type host in Pt‐SP‐tCz remains fully capable of forming an exciplex with the co‐doped n‐type host in the emitting layer. As a result, an additional *zero‐radius intramolecular energy transfer in the exciplex* (ZETPLEX) emerges, enabling the direct transfer of exciplex excitons to the Pt center with essentially no spatial loss. The combination of ZRIET and ZETPLEX produces an unprecedentedly high Förster energy transfer (FRET) rate of 3.64 × 10^8^ s^−1^ and recombination coefficient of 1.12 × 10^−7^ cm^3^ s^−1^ unattainable in conventional exciplex systems. As a result, the 2‐component‐EML PhOLED achieved an external quantum efficiency (EQE) of 23.6%, outperforming the EQE of the corresponding optimized 3‐component‐EML device.

This molecular‐integration strategy overcomes two long‐standing rate‐limiting processes in blue PhOLEDs—polaron recombination and exciton transfer—without relying on complex EML engineering. The result is a deep‐blue OLED that simultaneously achieves high efficiency, enhanced colour purity, and operational stability comparable to its multi‐component counterparts. Our work establishes molecular fusion as a generalizable design paradigm with the potential to reshape emitter and host engineering for next‐generation blue OLEDs.

## 2 Results and Discussion

### 2.1 Molecular Design and Synthesis Process

**FIGURE 1:**
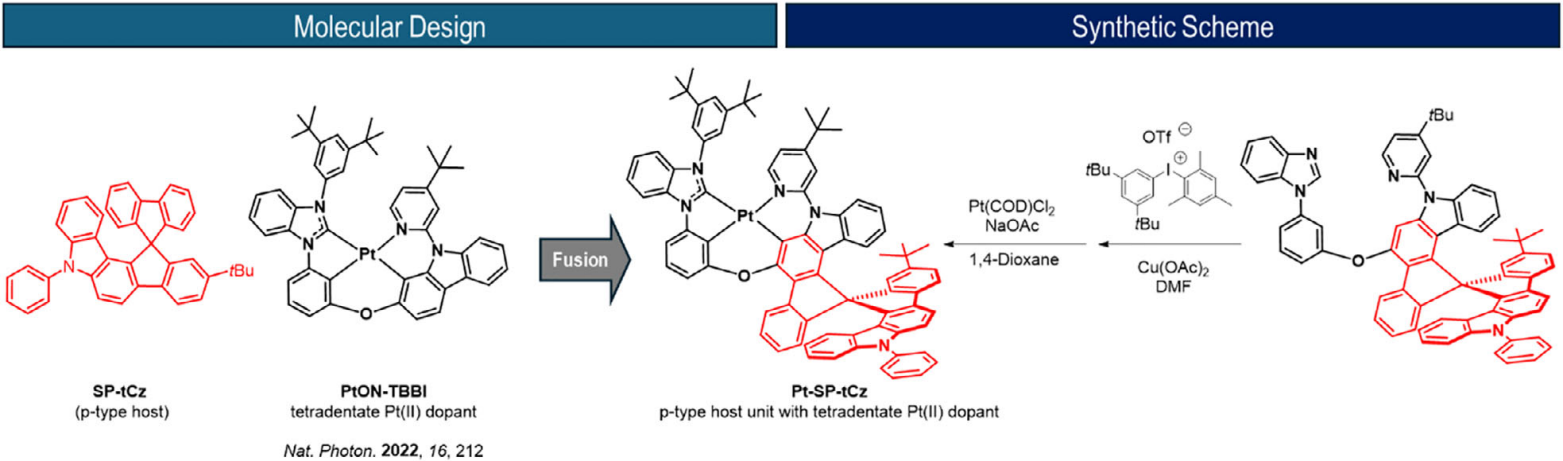
Molecular design and synthetic scheme of Pt‐SP‐tCz.

The molecular architecture of Pt‐SP‐tCz was designed to simultaneously achieve spatial proximity and electronic independence between the host and dopant subunits. For the construction of the ZETPLEX mechanism, a novel p‐type host, SP‐tCz, and a fused emitter, Pt‐SP‐tCz, were strategically designed and synthesized. Pt‐SP‐tCz was synthesized by integrating SP‐tCz with the tetradentate Pt(II) dopant, Pt‐SPCz. The synthesis process of the SP‐tCz and Pt‐SP‐tCz is shown in Figure 1. Detailed synthetic procedures are provided in the “Synthesis” section of the Supporting Information (SI). The chemical structures of SP‐tCz and PT‐SP‐tCz were confirmed by ^1^H and ^13^C nuclear magnetic resonance (NMR) and high‐resolution mass spectrometry (HR‐MS), as shown in Figures S1–S6.

### 2.2 Computational Analysis

Based on the optimization with ωB97xD functional, the highest occupied and lowest unoccupied molecular orbital (HOMO and LUMO) energies of Pt‐SP‐tCz, computed via single‐point calculation with the B3LYP functional, are −4.933 and −1.513 eV, respectively, which are slightly downshifted compared to those of Pt‐SPCz. (−4.905/−1.466 eV). In Figure S7, the frontier molecular orbital distributions were compared. The detailed information of the TDDFT calculation can be found in the “Computational details” section of the SI. By introduction of tCz unit at the spiro unit, the T_1_ energy of Pt‐SP‐tCz is calculated to be 2.661 eV, which is very similar to that of Pt‐SPCz (2.669 eV) in toluene medium, indicating that the chemically bonded tCz has a negligible effect on the emission energy [17]. The radiative decay rate (*k_r_*) from T_1_ to S_0_ states is computed to be 7.68 × 10^5^ s^−1^ with a <S_0_|H_SOC_|T_1_> of 217.81 cm^−1^. To ascertain whether tCz unit can function as a host, the S_n_ and T_n_ (n>1) states were further optimized, and the natural transition orbital (NTO) calculations were performed. As shown in Figure S8, the locally excited state solely contributes to the electronic structure in the S_7_, S_8,_ T_3_, and T_4_ states.

Specifically, the hole and electron NTOs for S_8_ and T_4_ states predominantly lie on the integrated tCz unit, mirroring the behavior of the independent SP‐tCz molecule. Although the energy gaps between S_8_/S_7_ and T_4_/T_3_ states are small, the NTOs of S_8_ and T_4_ states exhibit completely distinguishable spatial distributions from those of S_7_ and T_3_ states, indicating that the effect of the vibronic coupling on the internal conversion is expected to be negligible. Therefore, the fused tCz unit may behave as an independent p‐type host.

### 2.3 Physicochemical Properties

The thermal properties of SP‐tCz and Pt‐SP‐tCz were evaluated using thermogravimetric analysis (TGA) and differential scanning calorimetry (DSC), as presented in Figure S9. The HOMO and LUMO energies of SP‐tCz, PT‐SPCz, and Pt‐SP‐tCz were experimentally determined using differential pulse voltammetry (DPV), as shown in Figure S10 and summarized in Table S1. The LUMO energies of Pt‐SPCz and Pt‐SP‐tCz were estimated from their optical bandgaps. These energy levels are nearly identical for Pt‐SPCz and Pt‐SP‐tCz, consistent with theoretical predictions. Notably, Pt‐SP‐tCz exhibits two distinct oxidation peaks in its DPV curve, with the second peak corresponding to the SP‐tCz, which confirms the complete molecular fusion of the two units in Pt‐SP‐tCz. Similar dual‐peak features have been reported for other fully‐fused systems comprising distinct host and dopant units [15, 16]. While Pt‐based dopants typically act as hole traps in the host matrix, the presence of two distinct oxidation peaks in Pt‐SP‐tCz suggests that, through its fused structure, the dopant functions as a transport channel rather than a trap, thereby enabling the formation of dual recombination sites [16, 18].

### 2.4 Photophysical Properties

**FIGURE 2:**
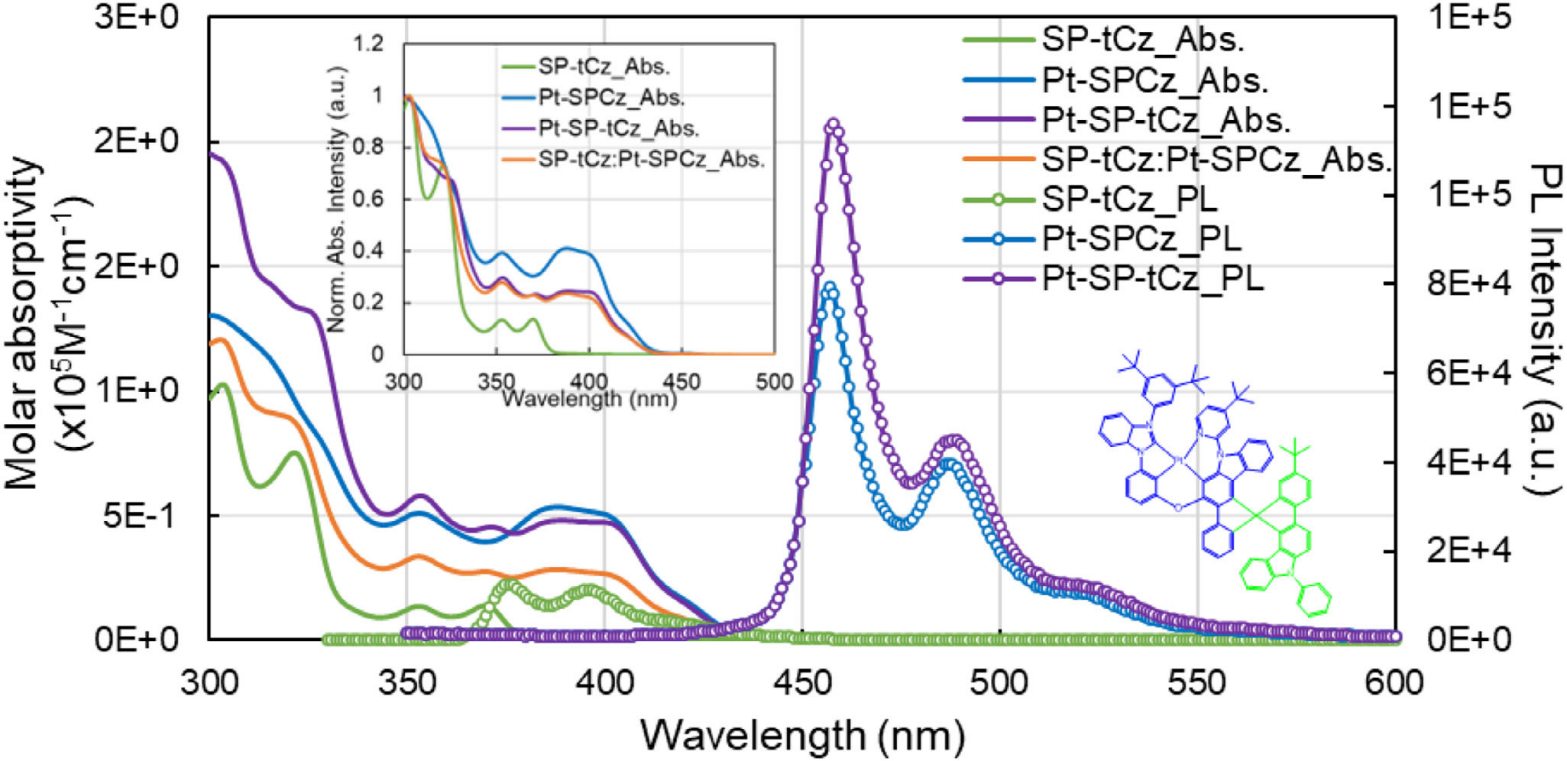
The UV–vis absorption spectra and PL spectra of SP‐tCz, Pt‐SPCz, Pt‐SP‐tCz, and a 50:50 mixture of SP‐tCz and Pt‐SPCz. The PL spectra were obtained by exciting the samples at 320 nm, corresponding to the absorption peak of SP‐tCz. The inset shows the normalized UV–vis absorption spectra of SP‐tCz, Pt‐SPCz, Pt‐SP‐tCz, and a 50:50 mixture of SP‐tCz and Pt‐SPCz. All samples were prepared in toluene solution a concentration of 2.0 × 10^−5^ M and measured at room temperature.

The UV–vis absorption and photoluminescence (PL) spectra of SP‐tCz, Pt‐SPCz, Pt‐SP‐tCz, and a mixture of SP‐tCz and Pt‐SPCz are presented in Figure 2. In the UV–vis absorption spectra, the intense peaks below 330 nm for SP‐tCz, Pt‐SPCz, and Pt‐SP‐tCz originate from the π–π* transition of the spiro moiety [19]. The absorption bands between 350 and 420 nm for Pt‐SPCz and Pt‐SP‐tCz are attributed to the metal‐to‐ligand charge transfer (^1^MLCT) transitions [20, 21]. The steady‐state absorption spectrum of Pt‐SP‐tCz features a clean superposition of the SP‐tCz band (below 330 nm) and the Pt‐SPCz band (350–420 nm), with no new absorption bands indicative of ground‐state perturbation and closely matches that of a physical mixture of SP‐tCz and Pt‐SPCz (inset). This result confirms that the fused architecture preserves the electronic and photophysical individuality of the two functional subunits without generating new hybridized states.

In the PL spectra, SP‐tCz, Pt‐SPCz, and Pt‐SP‐tCz were excited at 320 nm, corresponding to the absorption peak of SP‐tCz. SP‐tCz exhibits a main peak at 378 nm, while Pt‐SPCz and Pt‐SP‐tCz show their main peaks at 459 nm and the second peaks from the ^3^MLCT transition [22]. Interestingly, despite the mixed absorption characteristics of both SP‐tCz and Pt‐SPCz units within Pt‐SP‐tCz, only the PL of the emitting subunit (Pt‐SPCz) is observed, even when excited at 320 nm. The PL characteristics of Pt‐SP‐tCz indicate that intramolecular energy transfer occurs from the integrated SP‐tCz unit to the Pt‐SPCz unit within the Pt‐SP‐tCz molecule, providing strong evidence for ZRIET. Moreover, the PL spectrum of Pt‐SP‐tCz exhibits a significantly higher intensity than that of Pt‐SPCz. At 330 nm excitation, the photoluminescence quantum yield (PLQY) of Pt‐SPCz and Pt‐SP‐tCz are 23.9% and 20.3%, respectively, indicating that the difference in PL intensity does not stem from PLQY. Such enhancement indicates the implementation of the ZRIET mechanism in Pt‐SP‐tCz, wherein the minimized energy donor‐acceptor distance effectively suppresses energy loss and maximizes energy transfer efficiency [15, 16]. Physical characteristics such as S_1_ and T_1_ energies, exciton decay times obtained from transient PL (TrPL) measurements, are presented in Figures S11, S12, and Table S1.

### 2.5 Electroluminescence Properties

To fully validate the ZETPLEX concept, we first conducted a rigorous investigation into the foundational photophysical dynamics. We verified the formation of an exciplex between the host subunit, SP‐tCz, and the validated n‐type host, SiTrzCz2 [23]. The photophysical properties of the newly proposed SP‐tCz:SiTrzCz2 system are shown in Figure S13. The PL spectra showed a significant red‐shifted emission compared to individual components, and the temperature‐dependent TrPL profiles confirmed the long‐lived, thermally activated excited state, thereby providing unambiguous evidence for the SP‐tCz:SiTrzCz2 exciplex formation [24, 25]. This was further translated to electro‐optical performance, where a non‐doped SP‐tCz:SiTrzCz2 device exhibited clear exciplex characteristics in the EL spectrum, as the materials used in the study and the EL characteristics of the non‐doped devices are shown in Figures S14 and S15, respectively. We note the performance limitation of this non‐doped device (Table S2) stems from the deliberately simplified molecular design of SP‐tCz (spiro moiety and elemental carbazole unit), a structure intentionally adopted to ensure facile integration into the final Pt‐SP‐tCz molecule. This initial step establishes the intrinsic chemical potential of the SP‐tCz unit to form the necessary charge‐transfer states for the ZETPLEX operation.

**FIGURE 3:**
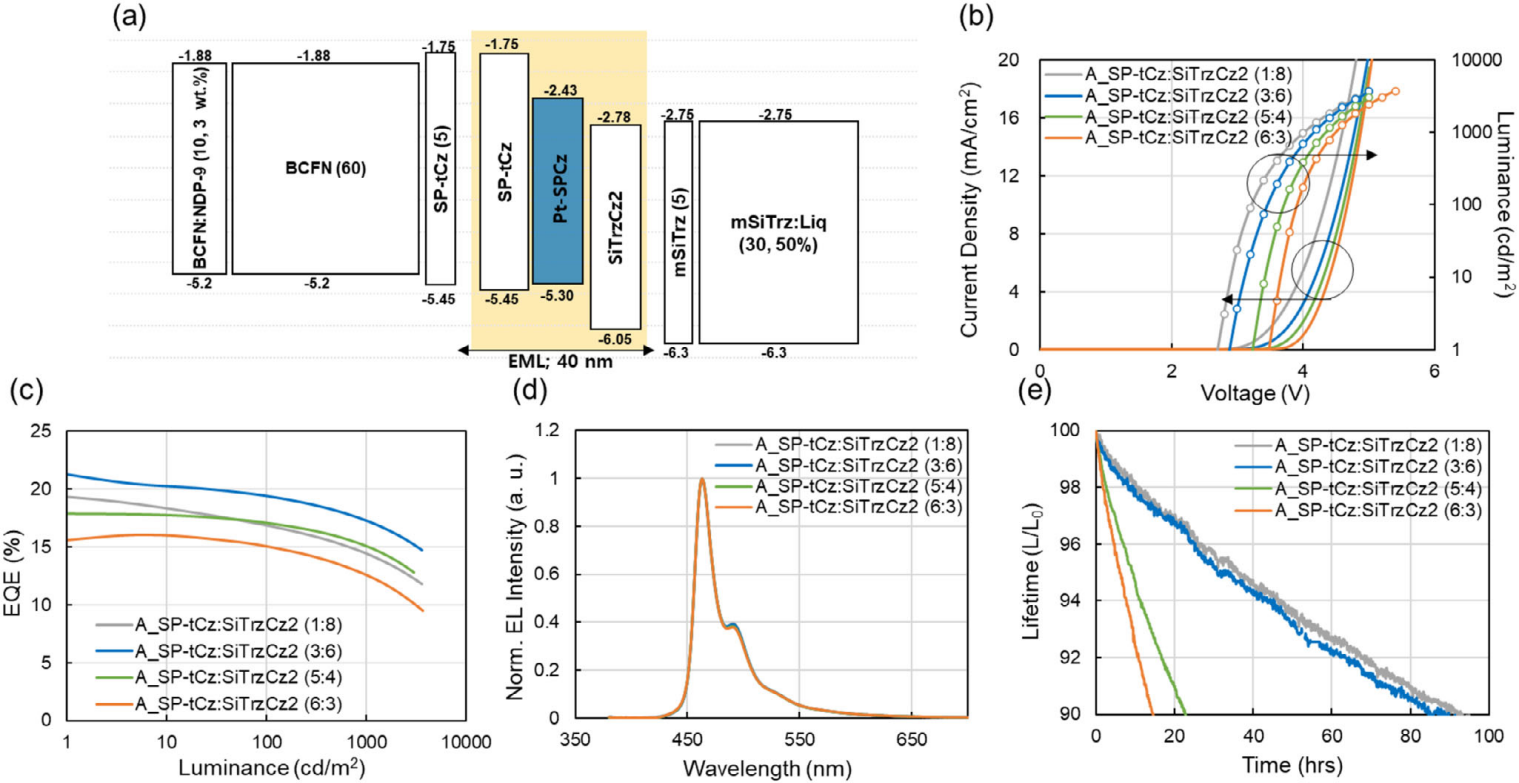
(a) Device structure, (b) *J–V–L* characteristics, (c) EQE curves, (d) EL spectra, and (e) Lifetime of the fabricated 3‐component EML PhOLEDs. The EMLs of A‐series devices were composed of SP‐tCz as the p‐type host, SiTrzCz2 as the n‐type host, and Pt‐SPCz as the dopant. To optimize the EML configuration, the relative concentrations of SP‐tCz and SiTrzCz2 were systematically varied. Lifetime was assessed based on LT_90_ values (The time required for luminance to decay to 90% of the initial value, measured at 500 nits).

As an early stage in developing the innovation EML architecture that incorporates new excitonic dynamics within a 2‐component system, A‐series PhOLEDs utilizing SP‐tCz were fabricated in a 3‐component configuration. To optimize the EML configuration, the device performances were compared by varying the composition ratio between SP‐tCz and SiTrzCz2 based on the device structure shown in Figure 3a. In Figure 3b, the operating voltage of the fabricated devices increases with the relative content of SP‐tCz. All devices exhibit EQEs exceeding 16%, with the SP‐tCz:SiTrzCz2 (3:6) device achieving a particularly high EQE of 21.9%. The A‐series devices exhibit EL spectra with a distinct emission peak at 463 nm, clearly corresponding to the characteristic emission of Pt‐SPCz. The operational lifetime of the 1:8, 3:6, 5:4, and 6:3 ratio devices is 90, 84, 24, and 15 h, respectively, showing a decreasing trend as the relative concentration of SP‐tCz increased. Interestingly, the device with an SP‐tCz:SiTrzCz2 ratio of 3:6 exhibited a higher efficiency than the 1:8 ratio device despite the comparable lifetimes. Therefore, among the fabricated A‐series devices, A_SP‐tCz:SiTrzCz2 (3:6), which exhibited the highest performance in terms of both efficiency and operational lifetime, was selected as the reference device.

**FIGURE 4:**
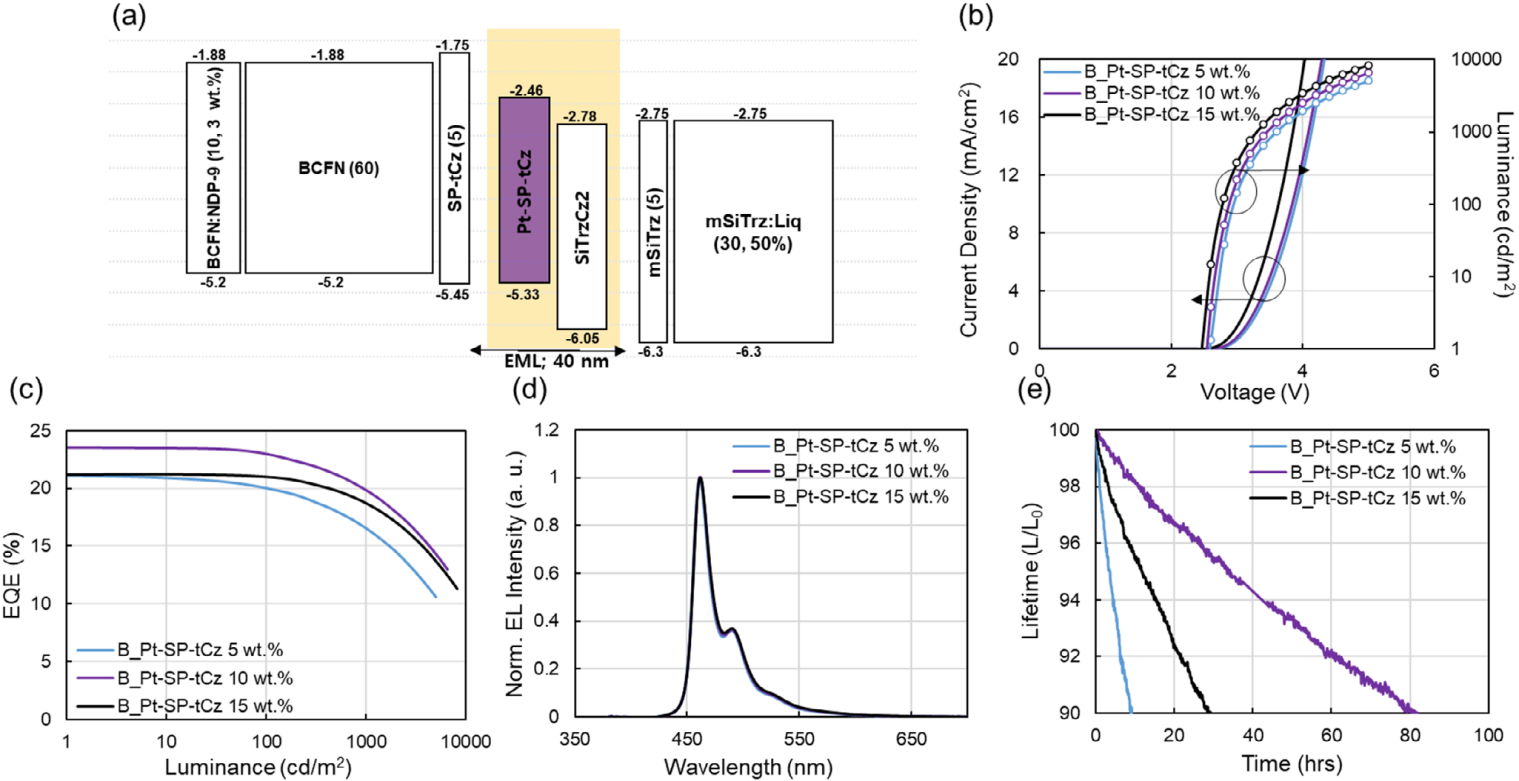
(a) Device structure, (b) *J–V–L* characteristics, (c) EQE curves, (d) EL spectra, and (e) Lifetime of the fabricated 2‐component EML PhOLEDs. The EMLs of B‐series devices are composed of the n‐type host SiTrzCz2 and Pt‐SP‐tCz. To optimize the 2‐component EML, the concentration of the incorporated Pt‐SP‐tCz was varied. Lifetime was assessed based on LT_90_ values (The time required for luminance to decay to 90% of the initial value, measured at 500 nits).

To pioneer an EML architecture that effectively embodies the ZRIET dynamics, we fabricated high‐performance blue PhOLEDs leveraging the integrated emitter Pt‐SP‐tCz, as shown in Figure 4a. The performance of these novel devices (B‐series: Pt‐SP‐tCz and SiTrzCz2) was benchmarked against conventional 3‐component devices (A‐series: Pt‐SPCz, SP‐tCz, and SiTrzCz2). While maintaining the same EML composition of the A‐series, the B‐series devices adopt a 2‐component system through an advanced architectural design. Optimized conditions were identified through systematic concentration variations. In Figure 4b, the operating voltage of the B‐series devices decreases with increasing concentration of Pt‐SP‐tCz. Interestingly, all B‐series devices exhibit “*significantly*” lower operating voltage than those of the A‐series devices. This voltage reduction is a critical indicator that the integrated Pt‐SPCz subunit, unlike in the conventional 3‐component configuration, does NOT function as a deleterious hole trap within the 2‐component EML. This behavior, further supported by DPV measurements, provides strong evidence for the presence of electrochemical dual channels in the Pt‐SP‐tCz molecule itself (Figure S10). In Figure 4c, the EQE of the devices is the lowest at 21.1% when the concentration of Pt‐SP‐tCz is 5 wt.%, reaches its highest value of 23.6% at 10 wt.%, and decreases again to 21.2% at 15 wt.%. When the Pt‐SP‐tCz concentration is too low, the SP‐tCz units within the Pt‐SP‐tCz molecules are also present in low amounts, which suppresses the formation of the exciplex with SiTrzCz2, leading to reduced efficiency. On the other hand, when the concentration of Pt‐SP‐tCz exceeds a certain level, concentration quenching of the Pt dopant may occur, resulting in a decline in device efficiency. Despite the simplification of the EML from a 3‐component to a 2‐component structure, the B_Pt‐SP‐tCz_10 wt.% device achieved a higher EQE than the conventional A_SP‐tCz:SiTrzCz2_(3:6) device. The EL spectra of the B‐series devices all exhibit the characteristic emission of Pt‐SPCz, indicating that Pt‐SP‐tCz follows the emission behavior of the intramolecularly integrated Pt‐SPCz unit. Moreover, the EL spectra of the B‐series devices yielded a deeper blue emission (461 nm of peak wavelength, CIEy = 0.149, 18 nm of full width at half maximum) with a minimized shoulder peak, signifying superior colour purity. The CIE diagrams of the fabricated PhOLEDs are presented in Figures S16–S18.

The operational lifetimes follow the same trend as the EQE, with the Pt‐SP‐tCz 5, 10, and 15 wt.% devices exhibiting lifetimes of 10, 82, and 31 h, respectively. Importantly, the B_Pt‐SP‐tCz_10 wt.% device maintained a comparable operational lifetime of 82 h, closely matching the 84 h of the A‐series device. Furthermore, it clearly surpasses the A‐series in both current and power efficiency (Figure S19), which we attribute to the significantly improved charge injection and transport enabled by the simplified, fused emitter‐host architecture. Additionally, PL and temperature‐dependent TrPL measurements also confirmed efficient exciplex formation even in the SP‐tCz:SiTrzCz2 (0.1:0.9) system, as shown in Figure S20. As shown in Figure S21, the reverse intersystem crossing (RISC) activation energies (E_a,RISC_) extracted from temperature‐dependent TrPL measurements are 20.1 meV for SP‐tCz:SiTrzCz2 (3:6) and 22.2 meV for SP‐tCz:SiTrzCz2 (1:9) films, respectively, which are nearly identical.

To further validate the functional role of the SP‐tCz unit, we fabricated a control 2‐component device using the isolated Pt‐SPCz emitter in SiTrzCz2, as shown in Figure S22. While this device showed a reduced operating voltage compared to A_SP‐tCz:SiTrzCz2_(3:6) device, it suffered a decline in operational lifetime, directly attributable to the severe charge imbalance caused by the absence of an effective p‐type host. In contrast, B_Pt‐SP‐tCz_10 wt.% maintained operational stability and high EQE. This evidence powerfully demonstrates that the SP‐tCz unit, integrated within the Pt‐SP‐tCz molecule, functions as an effective hole‐transporting moiety. These compelling results robustly confirm the effective implementation of the *ZETPLEX* mechanism. The ZETPLEX architecture successfully integrates efficient *ZRIET* with superior charge management within a single molecular entity, leading to simultaneous high efficiency, low driving voltage, and stable operation in a simplified EML. The performances of all fabricated PhOLEDs are summarized in Table S3.

### 2.6 Dual Recombination Sites and Ultrafast Energy Transfer

To gain a profound understanding of the exceptional performance delivered by the novel EML architecture with the ZETPLEX mechanism, we transitioned from macroscopic device metrics to a rigorous investigation of the underlying photophysical and electrochemical dynamics. The mechanistic superiority of the ZETPLEX system was first probed through steady‐state PL analysis. The PL spectra for the EML films of A_SP‐tCz:SiTrzCz2_(3:6) and B_Pt‐SP‐tCz_10 wt.% devices were collected under excitation at the specific absorption peak of the exciplex, as shown in Figure S23. The PL spectrum of the B_Pt‐SP‐tCz_10 wt.% film exhibits a distinct emission from the Pt‐SPCz dopant unit, which is identical to the emissive origin observed in the A_SP‐tCz:SiTrzCz2_(3:6) film. This observation provides the first crucial evidence of the ZETPLEX mechanism: an exciplex is effectively formed between the intramolecular SP‐tCz unit and the SiTrzCz2 host, followed by a near‐instantaneous energy transfer to the Pt‐SPCz emitting core via the ZRIET mechanism. This fused molecular design facilitates a radical simplification of the exciplex‐dopant system while maintaining, and even surpassing, the high‐performance benchmarks of conventional ternary systems.

The electrochemical advantage of this integration was further validated through the extraction of ideality factors for both the A_SP‐tCz:SiTrzCz2_(3:6) and B_Pt‐SP‐tCz_10 wt.% devices, as described in Figure S24. The B_Pt‐SP‐tCz_10 wt.% device exhibits a significantly lower ideality factor compared to the A‐series counterpart. This reduction is a definitive indicator of a substantial decrease in trap‐assisted recombination sites, suggesting that Langevin‐type recombination becomes the dominant pathway in the Pt‐SP‐tCz‐based EML [26]. Such findings are perfectly consistent with the enhanced charge injection and transport characteristics previously noted and provide strong macroscopic support for the existence of electrochemical dual channels within the Pt‐SP‐tCz molecule.

**FIGURE 5:**
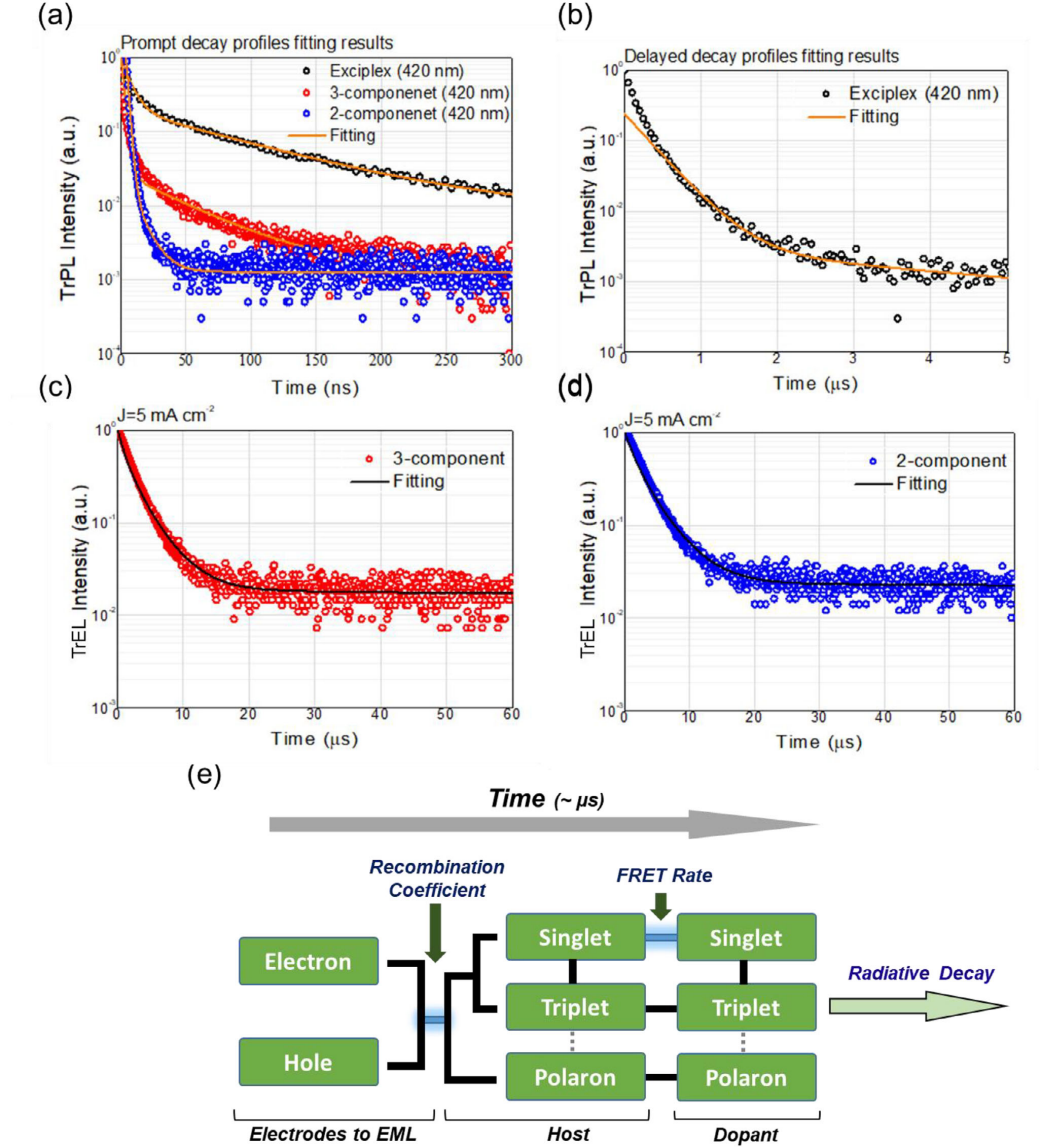
Polaron and exciton dynamics. The fitting results of (a) prompt and (b) delayed TrPL profiles of exciplex, 3‐component, and 2‐component EML films. The fitted TrEL profiles of (c) 3‐ component TrEL profile and (d) 2 component TrEL profile measured at current density of 5 mA cm^−2^. (e) Kinetic view of the electroluminescent process of phosphorescent OLEDs. Two main rate‐limiting processes are improved in this work.

PL and ideality factor analysis revealed clear photophysical and electrochemical differences between the conventional 3‐component exciplex‐based and the novel ZETPLEX‐based EMLs. To reach the core of this study, we conducted a comprehensive quantitative analysis of exciton (photophysical) and polaron (electrochemical) dynamics. We employed a sophisticated numerical modeling approach to fit the TrPL data from the films and transient EL (TrEL) data from the operational devices. The detailed calculation procedure is provided in “Calculation of Polaron and Exciton Dynamics” in the Supporting Information. The experimental and fitted TrPL data are presented in Figure S25 as well as in Figure 5a,b. Our numerical model successfully reproduced the reduction of the apparent exciton decay lifetime in the integrated systems compared to the host‐only control. From this modeling, we extracted the fundamental rate constants, which are summarized in Tables S4 and S5. Furthermore, the TrEL profiles and their corresponding numerical fits are displayed in Figure 5c,d, with the comprehensive results of the TrPL and TrEL analysis summarized in Table 1.

**TABLE 1:** Summarized TrPL. TrEL fitting parameters.

	τ_PF_ ^a^ (ns)	*k_PF_* ^b^ (× 10^7^ s^−1^)	*k*_*FRET*,*H*_ ^c^ (× 10^7^ s^−1^)	*k*_*ISC*.*H*_ ^d^ (× 10^6^ s^−1^)	*k*_*RISC*.*H*_ ^e^ (× 10^2^ s^−1^)	$k_{nr.H}^{T}$ ^f^ (× 10^2^ s^−1^)	*k*_*TT*.*H*_ ^g^ (× 10^−14^ cm^3^·s^−1^)	*k*_*TP*.*H*_ ^h^ (× 10^−10^ cm^3^·s^−1^)	*k*_*TT*.*D*_ ^i^ (× 10^−10^ cm^3^·s^−1^)	γ^j^ (× 10^−8^ cm^3^ s^−1^)
3‐component	18.1	5.52	4.03	1.21	1.69	3.43	1.03	1.82	3.60	3.22
2‐component	2.64	37.9	36.4	1.94	3.57	2.67	1.00	0.44	1.49	11.2

^a^ Prompt fluorescent time;

^b^ Prompt fluorescence rate constant;

^c^ FRET rate coefficient;

^d^ ISC rate of host;

^e^ RISC rate of host;

^f^ Nonradiative triplet exciton decay of host;

^g^ TTA rate of host;

^h^ TPA rate of host;

^i^ TTA of dopant;

^j^ recombination coefficient

The EL process in OLEDs is a complex sequence of sub‐microsecond events: polaron recombination, host‐to‐dopant energy transfer, and radiative decay [27, 28]. The kinetic processes are visualized in Figure 5e. Polaron recombination and FRET rate have been identified as the primary rate‐limiting bottlenecks. Simultaneously enhancing both processes has remained a “*Holy Grail*” in the field, as they originate from different physical principles.

**FIGURE 6:**
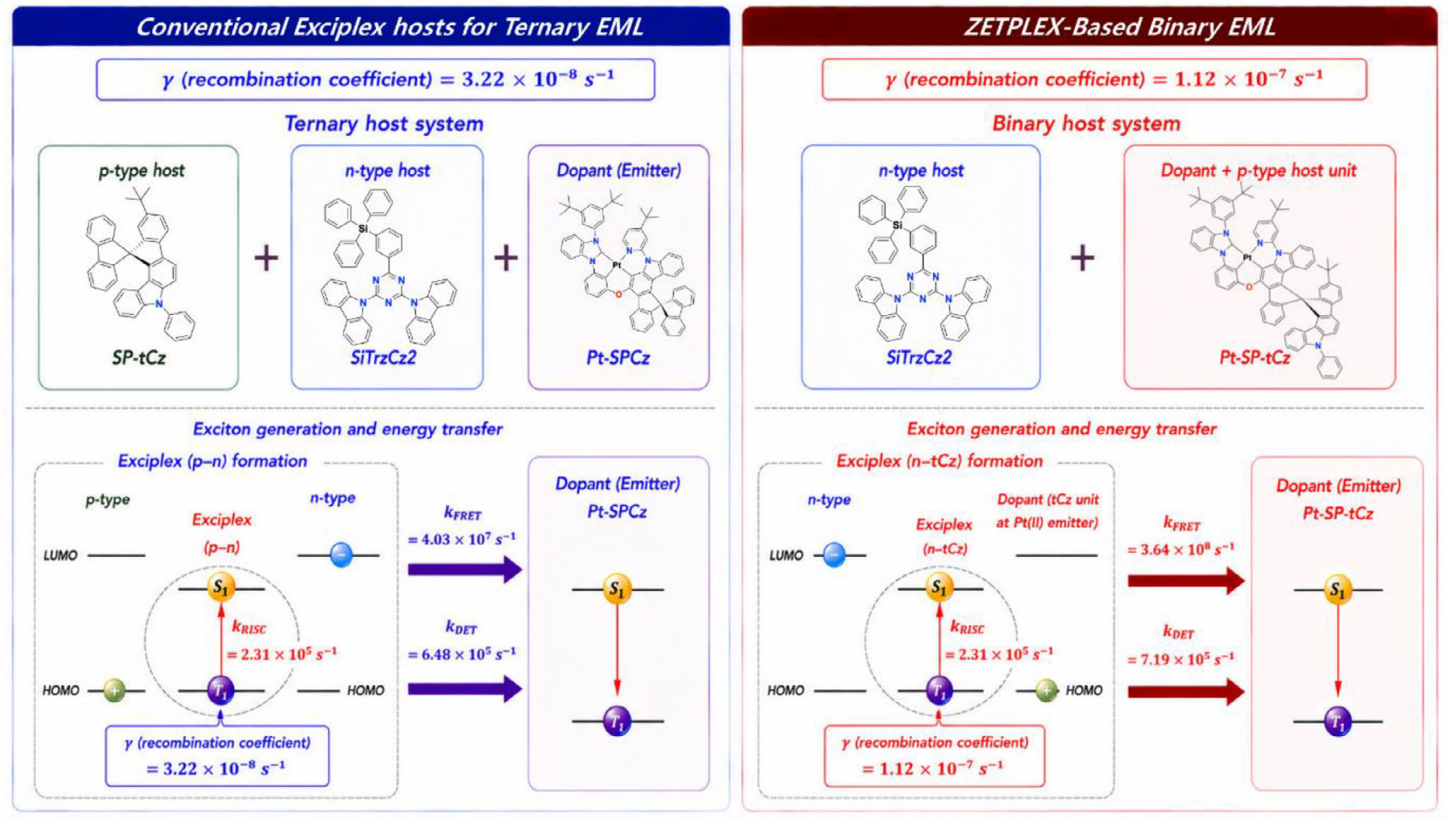
The schematic diagram of the polaron and exciton dynamics in the 3‐component conventional exciplex‐based and 2‐component ZETPLEX‐based EMLs.

However, the numerical analysis of the 3‐component exciplex‐based and the 2‐component ZETPLEX‐based EMLs, as illustrated in Figure 6, reveals a discovery of profound significance. Among all extracted parameters, the FRET rate constant, *k*_*FRET*,*H*_ exhibited a transformative enhancement. Specifically, *k*_*FRET*,*H*_ value was 4.03 × 10^7^ s^−1^ for 3‐component exciplex system but surged to an unprecedented 3.64 × 10^8^ s^−1^ for the 2‐component ZETPLEX system. This 9 fold acceleration represents a profound leap in exciton dynamics, achieving a record‐level value that has never been documented in the literature for blue organic semiconductors [12, 18, 29, 30, 31, 32]. This extraordinary rate is physically underpinned by the minimized intermolecular distance inherent to the fused architecture. As shown in Figure S26, the radial distribution functions (RDFs) for the Pt‐SP‐tCz/n‐type host system confirm that the exciplex is formed in far greater proximity to the Pt(II) complex (8.4 Å) than in the 3‐component mixture (9.0 Å). The chemically bonded tCz unit ensures that the exciplex formation site is localized within a minimal distance from the emitting core, thereby confirming the bifunctionality of Pt‐SP‐tCz as an emitter and a p‐type host.

Even more remarkably, the recombination coefficient, which governs the rate of polaron‐to‐exciton conversion, improved by 3.5 times, rising from 3.22 × 10^−8^ cm^3^ s^−1^ in the 3‐component system to 1.12 × 10^−7^ cm^3^ s^−1^ in the ZETPLEX‐based EML. This value is virtually unheard of in conventional organic semiconductor systems. This result irrefutably confirms the successful implementation of dual recombination sites within the Pt‐SP‐tCz molecule. By providing two distinct HOMO levels, as evidenced by the DPV measurements mentioned in Figure S10, the integrated molecular design creates dual electronic channels that facilitate a much more efficient encounter between electrons and holes.

In‐depth analysis of polaron and exciton dynamics demonstrates that the rational molecular integration of Pt‐SP‐tCz has effectively resolved two long‐standing challenges in OLED physics simultaneously. The kinetic parameters extracted from the simplified EML provide a coherent and powerful explanation for the superior charge injection, transport, and the record‐high FRET and recombination coefficient observed in our devices. These findings highlight that the ZETPLEX mechanism represents a fundamental advancement in EML engineering, pushing the limits of both polaronic and excitonic processes to levels previously deemed unreachable in the field of blue phosphorescence.

## 3 Conclusion

This study introduces a transformative molecular fusion strategy, integrating a p‐type host and Pt(II)‐dopant into Pt‐SP‐tCz. This architecture establishes dual recombination sites and an unprecedented FRET rate of 3.64 × 10^8^ s^−1^ via the ZETPLEX mechanism, achieving a superior EQE of 23.6% in blue PhOLEDs. By simultaneously optimizing polaron recombination and exciton transfer, this integrated design transcends long‐standing bottlenecks. Our findings provide a definitive, high‐performance platform for the next generation of blue OLED technology, mastering both photophysical and electrochemical dynamics within a single molecular entity.

## Author Contributions

**Garam Han**: methodology, investigation. **Eun Bi Kim**: investigation, methodology. **Bubae Park**: conceptualization, writing – original draft, methodology. **You Na Song**: writing – original draft, writing – review and editing, and conceptualization. **Wan Pyo Hong**: conceptualization, writing – original draft, methodology, writing – review and editing. **Hyung Youn Oh**: conceptualization, methodology, writing – original draft, writing – review and editing, funding acquisition. **Sunwoo Kang**: conceptualization, writing – original draft, methodology, writing – review and editing. **Jae‐Min Kim**: conceptualization, methodology, writing – review and editing, writing – original draft. **Junseop Lim**: methodology, investigation. **Taekyung Kim**: conceptualization, methodology, funding acquisition, writing – original draft, writing – review and editing, project administration.C

## Conflicts of Interest

The authors declare no conflict of interest.

## Supporting information




**Supporting File**: advs76331‐sup‐0001‐SuppMat.docx.

## Data Availability

The data that support the findings of this study are available from the corresponding author upon reasonable request.
