# Supplementary material for: Efficient Polaron Recombination and Fast Energy Transfer in a Deep Blue Phosphorescent Pt(II) Complex via Covalently Fused p‐Type Host
Source: Adv Sci (Weinh). 2026 Jun 28:e76331. Online ahead of print. doi: 10.1002/advs.76331 (PMC13336381; doi:10.1002/advs.76331)
Supplement: Supplementary file 1 — Supporting File: advs76331‐sup‐0001‐SuppMat.docx. [file ADVS-9999-e76331-s001.docx]

***Supporting information***

**Efficient Polaron Recombination and Fast Energy Transfer in a Deep Blue Phosphorescent Pt(II) Complex via Covalently Fused p-Type Host**

You Na Song^1^, Bubae Park^2^, Garam Han^2^, Eun Bi Kim^2^, Junseop Lim^3^, Wan Pyo Hong^4*^, Sunwoo Kang^5*^, Jae-Min Kim^6*^, Hyung Youn Oh^2*^, and Taekyung Kim^1*^

^1^Department of Chemical Engineering, Kyung Hee University, Yongin, Gyeonggi, 17104, Republic of Korea

^2^LORDIN, Hwaseong, Gyeonggi 18469, Republic of Korea

^3^Humboldt Centre for Nano- and Biophotonics, Institute for Light and Matter, Department of Chemistry, University of Cologne, 50939, Köln, Germany

^4^Department of Chemistry, Gachon University, Seongnam, Gyeonggi, 13120, Republic of Korea

^5^Department of Chemistry, Dankook University, Cheonan, Chungnam, 31116, Republic of Korea

^6^Department of Advanced Materials Engineering, Chung-Ang University, Anseong, Gyeonggi, 17546, Republic of Korea

Email: [wphongw@gachon.ac.kr](mailto:wphongw@gachon.ac.kr), [sunwoo.kang@dankook.ac.kr](mailto:sunwoo.kang@dankook.ac.kr), [jmkim52@cau.ac.kr](mailto:jmkim52@cau.ac.kr),

[ohygo@lordin.net](mailto:ohygo@lordin.net), and [taekyung.kim@khu.ac.kr](mailto:taekyung.kim@khu.ac.kr)

**Computational details**

Density functional theory (DFT) and time-dependent DFT (TDDFT) calculations were performed with ωB97xD functional with with LANL2DZ for Pt and Pople’s triple zeta potential with double polarization functions (6-311G**) for C, H, N, and O, as implemented in a suite of Gaussian 16 program.^[1]^ The optimally tuned ωB97xD (ω^*^B97xD) in conjunction with Tamm-Dancoff Approximation (TDA)^[2]^ were conducted to quantitatively obtain the photophysical properties of Pt-SP-tCz. Referred to the previous reports^[3]^, the optimal ω values of Pt-SP-tCz were set to be 0.098 Bohr^-1,^ which was determined at the minimum point of *J^2^*(ω) as a function of ω. The polarized continuum model (PCM) calculations were further conducted to consider excited-state energies in the toluene medium. The scalar relativistic zero-order regular approximation (ZORA) Hamiltonian was utilized to obtain the spin-orbit coupling (SOC) constant between S_0_ and T_1_ states, as implemented in the suite of ORCA 6.0.^[4]^ The SOC constants of <S_0_|H_SOC_|T_1_> were calculated at the optimized T_1_ structures with M062X/def2-TZVP level of theory. The radiative decay rate was derived from Einstein's spontaneous emission equation.^[5]^

Molecular dynamics (MD) simulations were performed, as implemented in the suite of Desmond package.^[6]^ The OPLS5 force field parameter was used to perform MD simulation. The convergence criteria of MD simulation were used as default values, as provided by the Desmond program. The initial bulk structures of 2-component and 3-component EMLs were built with a total of 256 molecules and consisted of Pt-SP-tCz (10 wt%): SiTrzCz2 and SP-tCz (6): SiTrzCz2 (4): Pt-SPCz (10 wt%). The multi-stage MD simulation protocols were processed by following four steps: (1) Brownian minimizations were initially conducted to gain reliable bulk structures. (2) NVT simulations at 1 atm and 300 K were carried out for 2 ns, (3) Simulated annealing simulations with NPT conditions (1 atm and 300 K) were carried out to describe the amorphous bulk. The temperature was gradually increased up to 1000 K with 7 ns, and then, cooled down at 300 K during 5 ns. (4) The NPT simulations at 1 atm and 300 K finally progressed during 7 ns.

**Nuclear Magnetic Resonance & High-resolution mass spectra**

^1^H and ^13^C nuclear magnetic resonance (NMR) spectra were recorded at room temperature using a Bruker 500 MHz spectrometer (VANCE III 500, Bruker). High-resolution mass spectra (HRMS) were obtained via fast atom bombardment (FAB) using a JMS-700 instrument (6890 series, JEOL).

**UV-vis Absorption**

UV–vis absorption spectra were recorded at room temperature using a UV–visible spectrophotometer (Agilent 8453). Sample solutions were prepared at a concentration of 2x10^-5^ M in toluene to minimize inner filter effects. Sample films were prepared by thermal evaporation onto quartz substrates with a thickness of 40 nm. Prior to measurement, all solutions were thoroughly purged with nitrogen to remove dissolved oxygen.

**Photoluminescence & Transient PL**

Photoluminescence (PL) and transient PL (TrPL) measurements were performed using a spectrofluorometer (FS5, Edinburgh Instruments). For solution-phase PL measurements, 4.0 mL of a 2 μM toluene solution was prepared and loaded into a quartz cuvette with a 1.0 cm path length. PL spectra were acquired at RT using 310 nm or 330 nm excitation pulse from a xenon lamp. TrPL measurements were carried out using a 375 nm pulsed laser to excite the samples, and the emission decays were collected via time-correlated single-photon counting (TCSPC, 1024 channels) using a picosecond pulsed source. The instrument response function (IRF) in this mode was on the order of a few nanoseconds.

**Differential Pulse Voltammetry Measurement**

Differential pulse voltammetry (DPV) was performed using an electrochemical analyzer (PGSTAT101, Autolab) to determine the HOMO and LUMO energy levels. Organic molecules were dissolved in nitrogen-purged dimethylformamide (DMF) containing 0.1 M tetrabutylammonium hexafluorophosphate (TBAPF₆) as the supporting electrolyte. A 10 mL aliquot of the solution was loaded into a quartz cell for measurement. The working, counter, and reference electrodes were all obtained from BASi (Bioanalytical Systems Inc.). DPV was conducted with a pulse width of 0.2 s, a pulse period of 0.5 s, and a step height of 25 mV. The ferrocenium/ferrocene (Fc⁺/Fc) redox couple was used as an internal reference. The HOMO and LUMO energy levels were calculated using the following equation:

HOMO/LUMO (eV) = 4.8 – E(Fc⁺/Fc) + E(ox/red),

where E(ox) and E(red) are the first oxidation and reduction potentials, respectively.

**Transient EL**

Transient electroluminescence (TrEL) was measured under a constant current density of 5 mA cm⁻². A rectangular pulse with a 1:1 aspect ratio and a total cycle time of 1.0 ms was applied using a function generator. To suppress EL spikes caused by residual charge carrier recombination during the turn-off period, a –4 V offset voltage was applied.

**OLED Fabrication & Characterization**

All devices were fabricated on indium-tin-oxide (ITO) substrates with a thickness of 150 nm. Prior to fabrication, the ITO substrates were subjected to sequential chemical cleaning via sonication in acetone, deionized water, and isopropyl alcohol for 15 minutes each. Following cleaning, substrates were dried in a convection oven at 120 °C for over 8 hours to ensure complete removal of residual solvents. All organic materials were deposited under high vacuum conditions (<2 × 10⁻⁷ Torr) after being loaded and outgassed for more than 24 hours. Device fabrication was conducted within the same vacuum environment. Deposition rates of organic layers and the aluminum (Al) cathode were maintained at approximately 1.0 Å/s and 2.0 Å/s, respectively, and were continuously monitored using a quartz crystal microbalance sensor. Completed devices were encapsulated inside a nitrogen-filled glove box with H₂O and O₂ levels below 0.1 ppm. Current density–voltage–luminance (*J–V–L*) characteristics and electroluminescence (EL) spectra were measured at room temperature using a source measurement unit (Keithley 2400) and a chroma meter (Konica Minolta CS-2000).

**Device structures**

The fabricated non-doped device’s structure is as follows: ITO (Anode, 150 nm) BCFN:p-dopant (3 wt.%, 10 nm) / BCFN (60 nm) / SiCzCz or SP-tCz (5 nm) / SiCzCz or SP-tCz:SiTrzCz2 (55:45 wt.%) (40 nm) /mSiTrz (5 nm) / mSiTrz:Liq (50 wt.%, 30 nm) / Liq (1.5 nm) / Aluminum (Cathode, 100 nm)

The fabricated A-series PhOLED’s structure is as follows: ITO (Anode, 150 nm) BCFN:p-dopant (3 wt.%, 10 nm) / BCFN (60 nm) / SP-tCz (5 nm) / SP-tCz:SiTrzCz2:Pt-SPCz (10:80:10 or 30:60:10 or 50:40:10 or 60:30:10 wt.%) (40 nm) /mSiTrz (5 nm) / mSiTrz:Liq (50 wt.%, 30 nm) / Liq (1.5 nm) / Aluminum (Cathode, 100 nm)

The fabricated B-series PhOLED’s structure is as follows: ITO (Anode, 150 nm) BCFN:p-dopant (3 wt.%, 10 nm) / BCFN (60 nm) / SP-tCz (5 nm) / SiTrzCz2:Pt-SP-tCz (95:5 or 90:10 or 85:15 wt.%) (40 nm) /mSiTrz (5 nm) / mSiTrz:Liq (50 wt.%, 30 nm) / Liq (1.5 nm) / Aluminum (Cathode, 100 nm)

The fabricated Pt-SPCz (2-component) device’s structure is as follows: ITO (Anode, 150 nm) BCFN:p-dopant (3 wt.%, 10 nm) / BCFN (60 nm) / SP-tCz (5 nm) / SiTrzCz2: Pt-SPCz (90:10 wt.%) (40 nm) /mSiTrz (5 nm) / mSiTrz:Liq (50 wt.%, 30 nm) / Liq (1.5 nm) / Aluminum (Cathode, 100 nm)

**Synthesis**

The P-4 ligand, corresponding to the eastern fragment of Platinum(II)[6-(1,3-Dihydro-3-(3,5-di-tert-butylphenyl)-2H-imidazol-2-ylidene-κC^2^)-1,2-phenylene-κC1]-oxy[10'-(tert-butyl)-5-(4-(tert-butyl)pyridin-2-yl-κN)-5'-phenyl-5H,5'H-12,12'-spirobi[indeno[1,2-c]carbazole-6,7-diyl-κC^1^] (Pt-SP-tCz), was synthesized from P-5, which was in turn obtained from the known carbazole P-6 through a sequence involving a standard Ullmann coupling, lithiation–borylation, and a Suzuki coupling reaction.


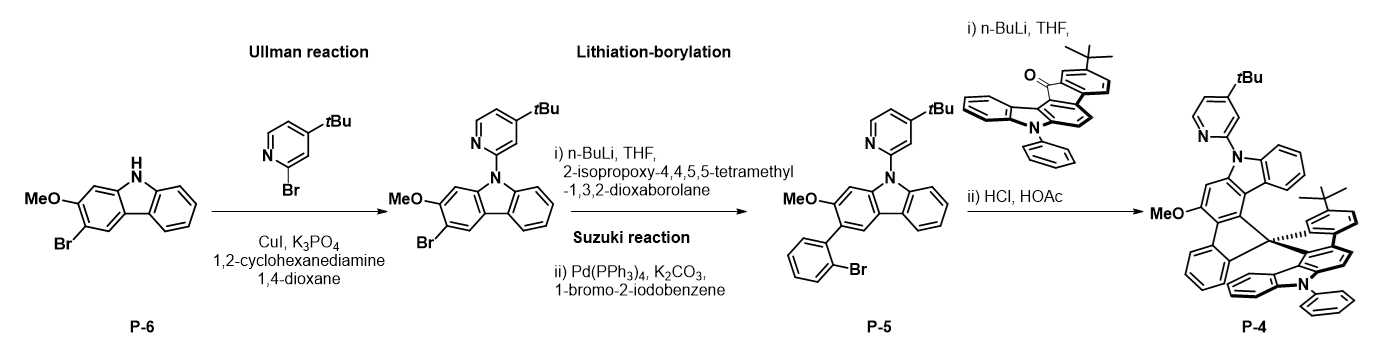


The two key ligands were then coupled via a C–O Ullmann reaction to afford the tetradentate ligand P-2. Subsequent phenylation gave P-1, and Pt-SP-tCz was finally obtained by metalation with dichloro(1,5-cyclooctadiene)platinum(II).

Synthesis of 10'-(*tert*-butyl)-5-(4-(*tert*-butyl)pyridin-2-yl)-7-methoxy-5'-phenyl-5*H*,5'*H*-12,12'-spirobi[indeno[1,2-*c*]carbazole] (P-4)

3-(2-bromophenyl)-9-(4-(*tert*-butyl)pyridin-2-yl)-2-methoxy-9*H*-carbazole (P-5) (53.3 g, 0.103 mol) was dissolved in dry THF (500 mL) and cooled down to -78 °C. A 2.5 M pentane solution of n-BuLi (45.32 mL, 0.113 mol, 1.1 eq) was then added dropwise to the solution at -78°C. The resulting mixture was stirred at the same temperature for one hour and 10-(*tert*-butyl)-5-phenylindeno[1,2-*c*]carbazol-12(5*H*)-one (37.22 g, 92.7 mmol) dissolved in dry THF (400 mL) was added dropwise. The reaction mixture was allowed to warm up to room temperature and stirred overnight. Absolute ethanol (20 mL) was added and the reaction mixture was concentrated under reduced pressure. The residue was dissolved in acetic acid (300 mL) and the solution was heated under reflux before concentrated hydrochloric acid (30 mL) was added. The resulting mixture was stirred under reflux overnight, then cooled down to room temperature and added to water at 0°C (1200 mL). The organic phase was extracted with dichloromethane and washed with a saturated aqueous solution of sodium thiosulfate. The organic layer was then dried over magnesium sulfate, filtrated and concentrated. The residue was purified by flash chromatography on silica gel. The product was recrystallized from DCM/hexane to give a colourless powder (33.68 g, yield: 46%). ^1^H NMR (400 MHz, CD_2_Cl_2_) δ 8.63 (d, *J* = 5.3 Hz, 1H), 8.40 (d, *J* = 7.7 Hz, 1H), 8.14 (d, *J* = 8.4 Hz, 1H), 7.93 (d, *J* = 8.0 Hz, 1H), 7.62 – 7.54 (m, 5H), 7.51 – 7.45 (m, 3H), 7.44 – 7.37 (m, 2H), 7.36 – 7.30 (m, 2H), 7.10 – 6.99 (m, 3H), 6.93 (t, *J* = 7.5 Hz, 1H), 6.79 (d, *J* = 8.0 Hz, 1H), 6.75 – 6.58 (m, 5H), 4.24 (s, 3H), 1.38 (s, 9H), 1.08 (s, 9H).

Finally, the P-4 ligand was converted to P-3. Using P-3 and 1-(3-bromophenyl)-1*H*-benzo[*d*]imidazole, the two main ligands were coupled via a C-O Ullman reaction, leading to the formation of the tetradentate ligand P-2. Subsequent phenylation produced P-1, and Pt-SP-tCz was synthesized through metalation using dichloro(1,5-cyclooctadiene)platinum(II).

Synthesis of 10'-(*tert*-butyl)-5-(4-(*tert*-butyl)pyridin-2-yl)-5'-phenyl-5*H*,5'*H*-12,12'-spirobi[indeno[1,2-*c*]carbazol]-7-ol (P-3)

P-4 (11.9 g, 15.0 mmol) was dissolved in DCM (175 mL) and added to the flask. The flask was stirred at 0 °C. Boron tribromide (7.56 g, 30.2 mmol) was dropwised into flask and stirred at 25 °C for 3 h. After that, the product was quenched with water and extracted using DCM/water and washed by aqueous ammonium chloride. After further purification by column chromatography using hexane:ethyl acetate (5:1) eluent, a solid (P-3) was obtained (7.9 g, 68%).  ^1^H NMR (400 MHz, CD_2_Cl_2_) δ 8.63 (d, *J* = 5.4 Hz, 1H), 8.31 (d, *J* = 7.6 Hz, 1H), 8.14 (d, *J* = 8.4 Hz, 1H), 7.93 (d, *J* = 8.0 Hz, 1H), 7.64 (s, 1H), 7.61 – 7.53 (m, 4H), 7.51 – 7.45 (m, 3H), 7.42 (dd, *J* = 8.1, 1.8 Hz, 1H), 7.36 – 7.33 (m, 2H), 7.28 (dd, *J* = 10.9, 4.2 Hz, 1H), 7.09 – 7.00 (m, 2H), 6.94 (ddd, *J* = 10.8, 8.3, 4.1 Hz, 3H), 6.83 (d, *J* = 8.0 Hz, 1H), 6.79 (d, *J* = 1.6 Hz, 1H), 6.74 – 6.59 (m, 4H), 1.37 (d, *J* = 4.7 Hz, 9H), 1.07 (d, *J* = 7.7 Hz, 9H).

Synthesis of ((trifluoromethyl)sulfonyl)-λ^1^-oxidane, 1-(3-((10'-(*tert*-butyl)-5-(4-(*tert*-butyl)pyridin-2-yl)-5'-phenyl-5*H*,5'*H*-12,12'-spirobi[indeno[1,2-*c*]carbazol]-7-yl)oxy)phenyl)-3-(3,5-di-*tert*-butylphenyl)-1H-benzo[*d*]imidazol-3-ium salt (P-1)

P-3 (11.2 g, 14.4 mmol), 1-(3-bromophenyl)-1*H*-benzo[*d*]imidazole (3.27 g, 12.0 mmol), CuI (0.82 g, 4.2 mmol), picolinic acid (12.25 g, 57.7 mmol) and K_3_PO_4_ (2.66 g, 21.6 mmol) were added to the flask and dissolved in DMSO (80 mL). The flask was stirred at 125 °C at 16 h. After that, the product was quenched with water and extracted using ethyl acetate/water and washed by aqueous ammonium chloride. After further purification by column chromatography using hexane:ethyl acetate (2:1) eluent, a pink powder (P-2) was obtained.

P-2 (4.5 g, 4.6 mmol), (3,5-di-*tert*-butylphenyl)(mesityl)iodonium trifluoromethanesulfonate (4.7 g, 8.0 mmol) and Cu(OAc)_2_ (0.10 g, 0.5 mmol) were added to the flask and dissolved in DMF (40 mL). The flask was stirred at 140 °C at 3 h. After that, filtered roughly through a short pad of silica and washed with acetone:DCM (1:5) eluent. The resulting product (P-1) was used in the next reaction without further purification.

Synthesis of Pt-SP-tCz

P-1 (2.5 g, 1.91 mmol), dichloro(1,5-cyclooctadiene)platinum(II) (0.79 g, 2.10 mmol) and sodium acetate (0.47 g, 5.74 mmol) were added to the flask and dissolved in DMF (38 mL). The flask was stirred at 120 °C at 12 h. After that, the product was extracted with DCM/water. A yellow solid was obtained and used without further purification. After further purification by column chromatography using hexane: DCM (5:1) eluent, a yellow powder was obtained. (0.51 g, yield 20%). ^11^H NMR (400 MHz, CD_2_Cl_2_) δ 8.95 (d, *J* = 7.7 Hz, 1H), 8.66 (d, *J* = 6.4 Hz, 1H), 8.24 (d, *J* = 8.3 Hz, 1H), 8.05 (d, *J* = 8.5 Hz, 1H), 7.83 (d, *J* = 8.1 Hz, 1H), 7.73 (d, *J* = 7.6 Hz, 1H), 7.66 (s, 1H), 7.61 – 7.43 (m, 9H), 7.42 – 7.34 (m, 5H), 7.31 (t, *J* = 7.5 Hz, 3H), 7.03 – 6.92 (m, 2H), 6.87 (dd, *J* = 11.4, 7.8 Hz, 3H), 6.68 (s, 1H), 6.63 (t, *J* = 7.5 Hz, 1H), 6.54 – 6.43 (m, 2H), 6.09 (dd, *J* = 6.4, 1.9 Hz, 1H), 1.62 – 0.84 (m, 36H); HRMS (FAB^+^) calcd for C83H72N5OPt [M+H]: 1349.5379, found 1349.5348.

Synthesis of 10'-(tert-butyl)-5'-phenyl-5'H-spiro[fluorene-9,12'-indeno[1,2-c]carbazole] (SP-tCz)

2-bromo-1,1'-biphenyl (2.3 g, 0.010 mol) was dissolved in dry THF (30 mL) and the solution was cooled to -78 °C. A 2.5 M pentane solution of n-BuLi (4.4 mL, 0.011 mol, 1.1 eq) was added dropwise at -78°C, and the resulting mixture was stirred at this temperature for 0.5 h. A solution of 10-(*tert*-butyl)-5-phenylindeno[1,2-*c*]carbazol-12(5*H*)-one (4.02 g, 0.01 mol) in dry THF (40 mL) was then added dropwise. The reaction mixture was allowed to warm to room temperature and stirred overnight. Absolute ethanol (2 mL) was added, and the mixture was concentrated under reduced pressure. The residue was dissolved in acetic acid (30 mL), and the solution was heated to reflux, after which concentrated hydrochloric acid (3 mL) was added. The mixture was refluxed overnight, cooled to room temperature, and poured into water (100 mL) at 0 °C. The aqueous phase was extracted with dichloromethane, and the combined organic layers were washed with saturated aqueous sodium thiosulfate, dried over magnesium sulfate, filtered, and concentrated. The residue was purified by flash chromatography on silica gel. The product was recrystallized from DCM/hexane to give a colourless powder (3.11 g, yield: 58%). ^1^H NMR (400 MHz, CDCl_3_) δ 8.07 – 7.99 (m, 3H), 7.90 (d, *J* = 8.4 Hz, 1H), 7.76 – 7.70 (m, 1H), 7.63 – 7.32 (m, 9H), 7.18 – 7.08 (m, 1H), 7.04 (td, *J* = 7.5, 1.3 Hz, 2H), 6.86 – 6.78 (m, 3H), 6.72 – 6.62 (m, 1H), 6.60 – 6.58 (m, 1H), 1.14 (s, 9H); HRMS (EI) calcd for C41H31N [M]^+^: 537.2457, found 537.2453.

**
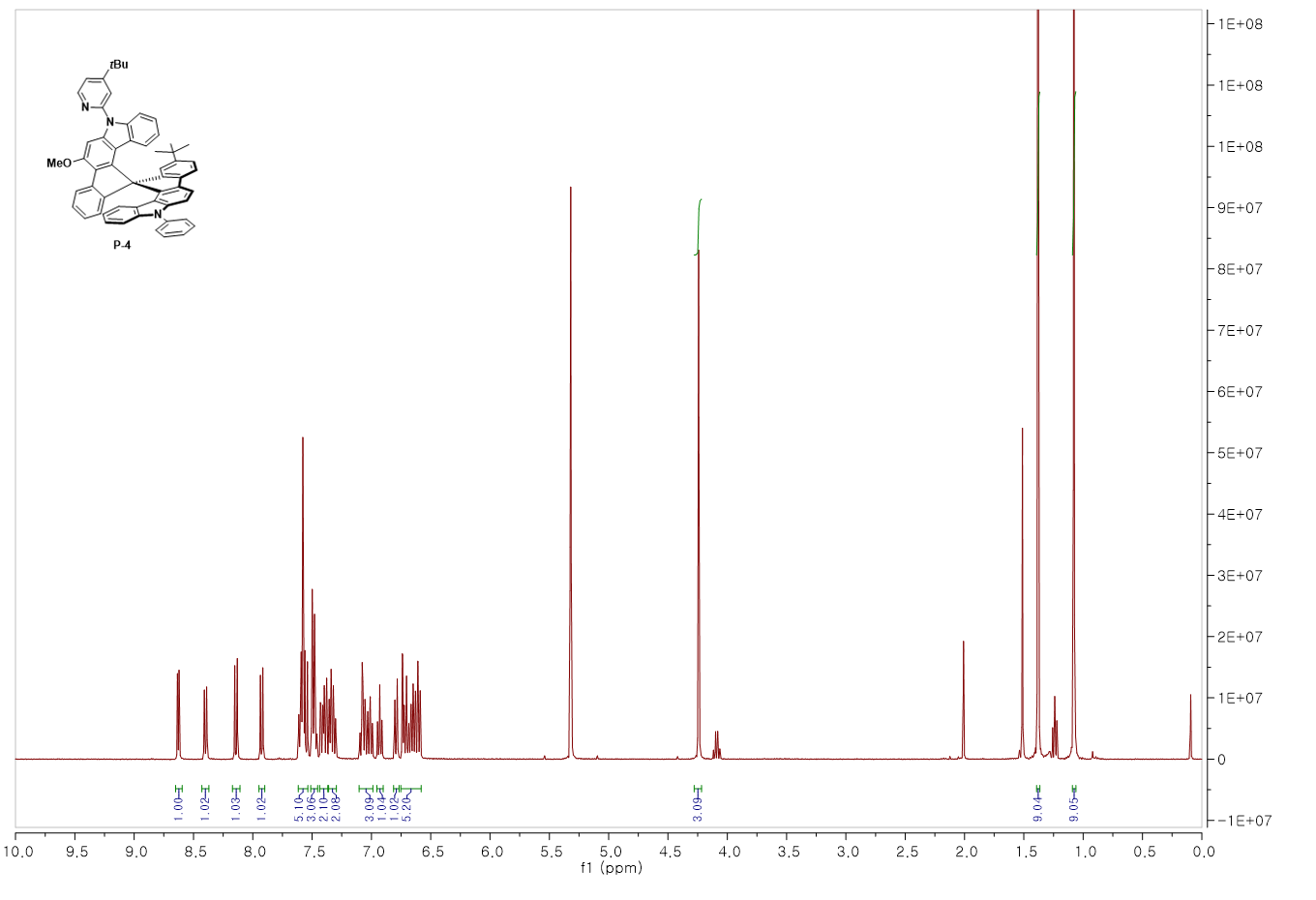
**

**Figure S1. ^1^H NMR of P-4.**

**
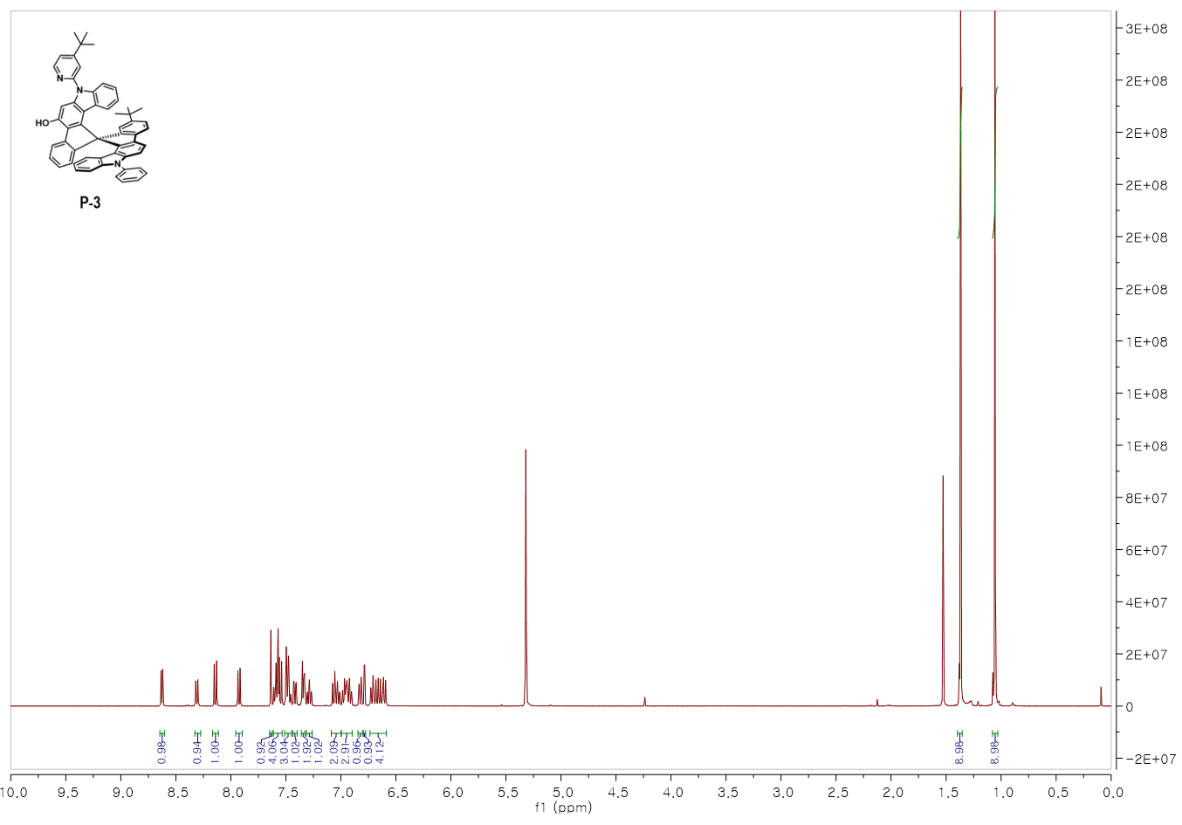
**

**Figure S2. ^1^H NMR of P-3.**

**
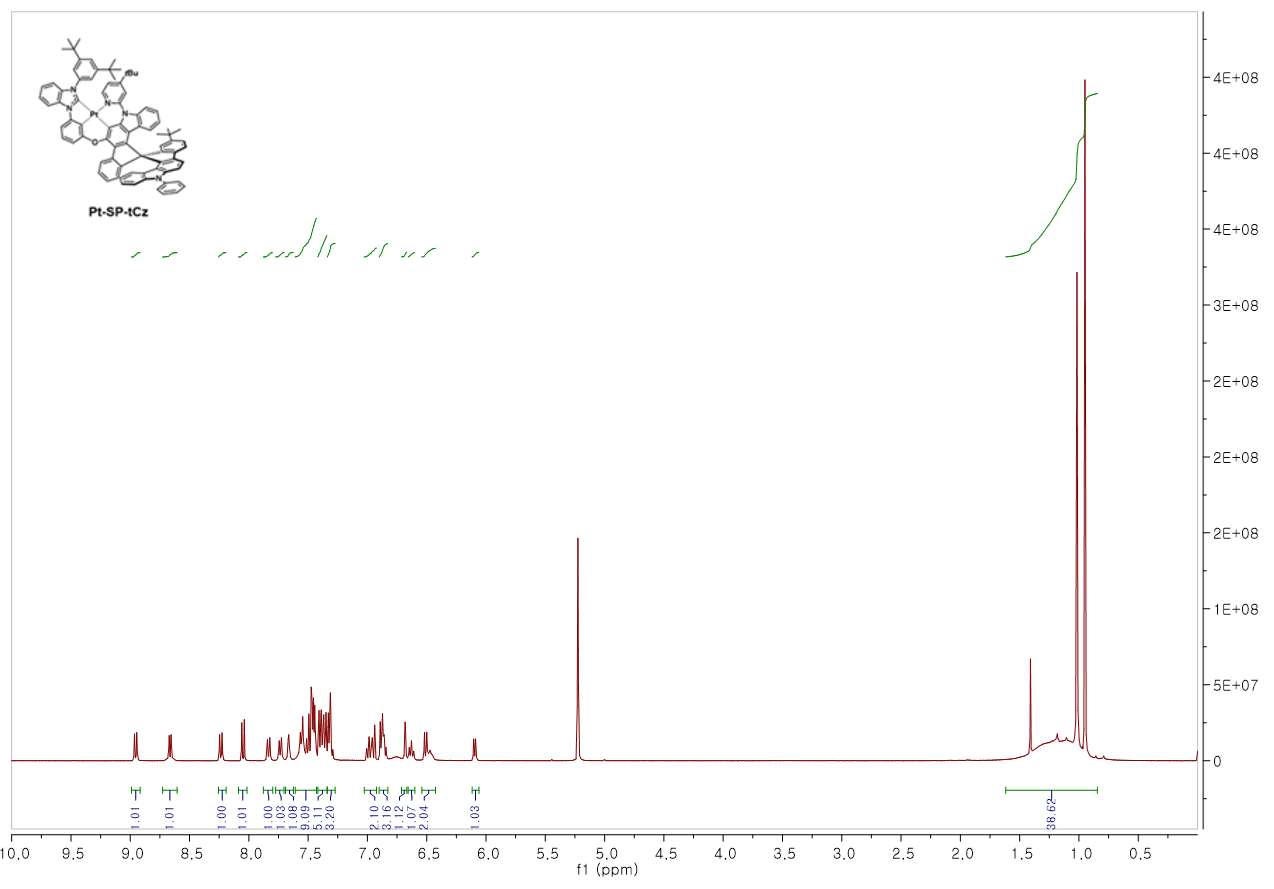
**

**Figure S3. ^1^H NMR of Pt-SP-tCz.**

**
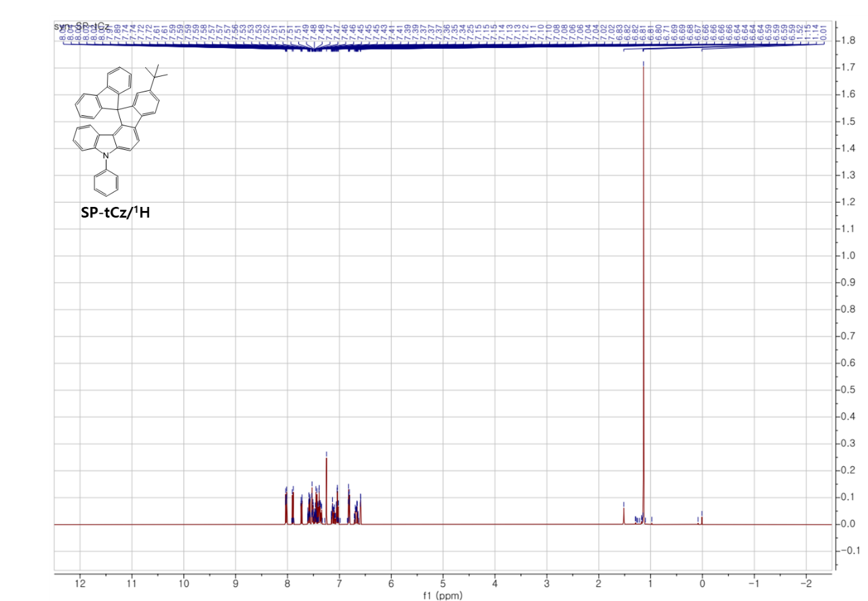
**

**Figure S4. ^1^H NMR of SP-tCz.**

**
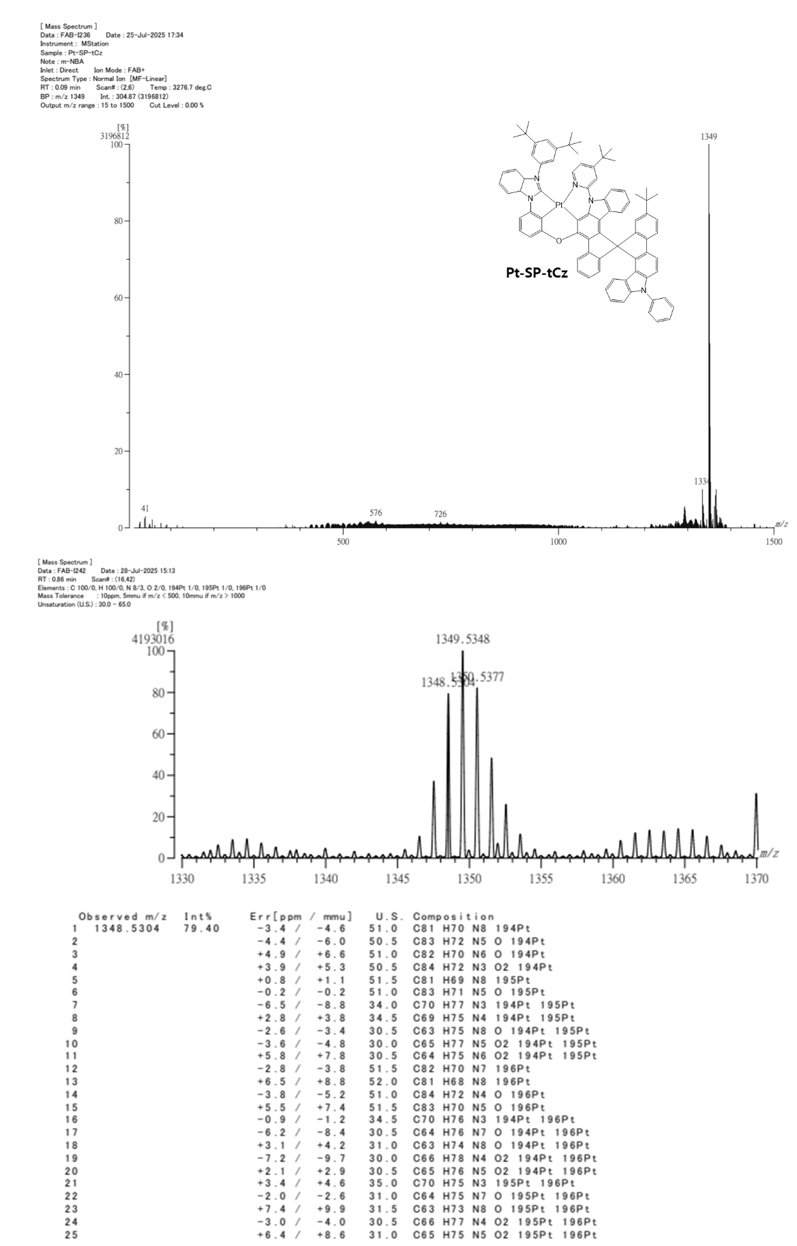
**

**Figure S5. HR-MS of Pt-SP-tCz.**

**
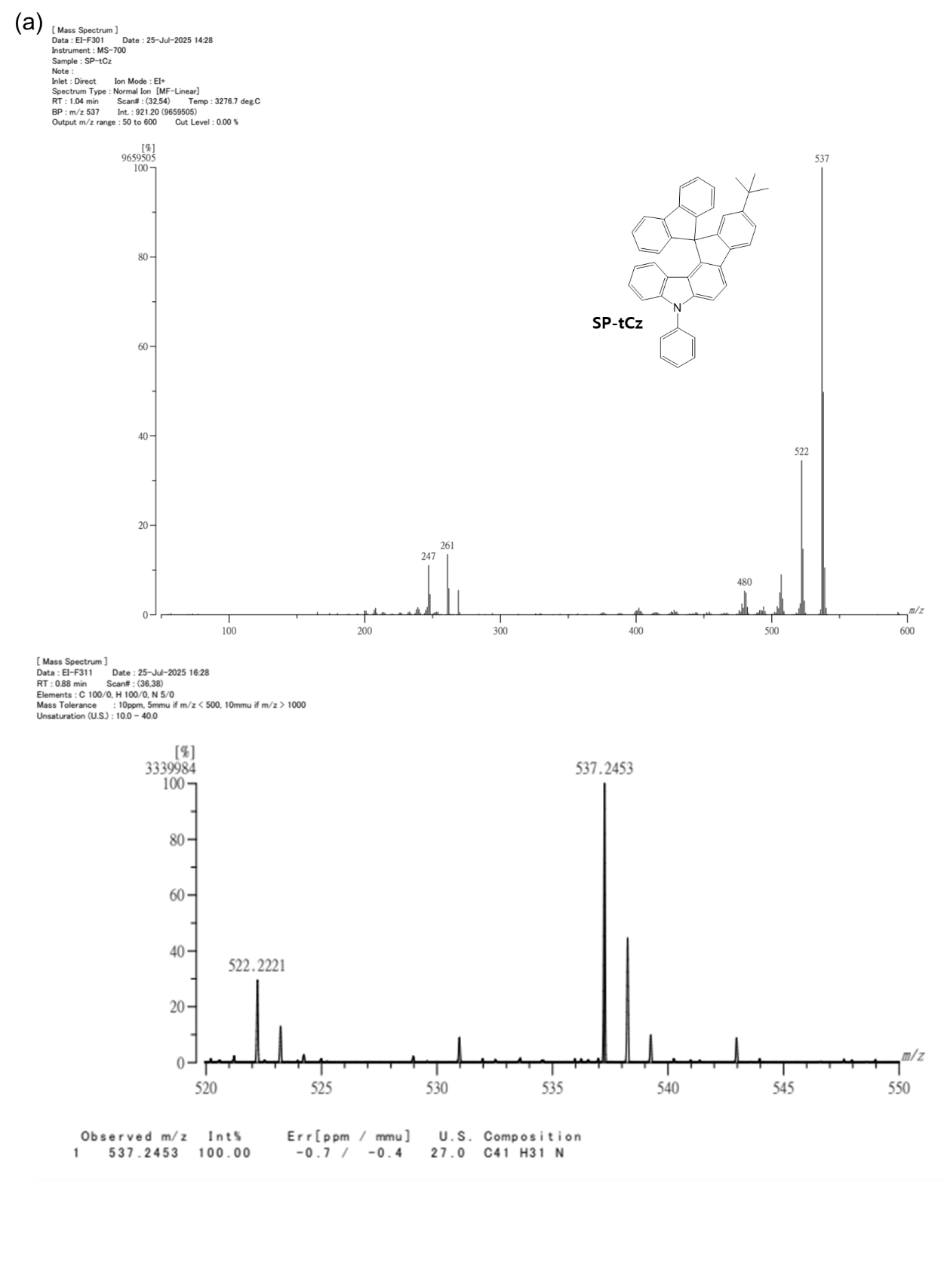
**

**Figure S6. HR-MS of SP-tCz.**

**
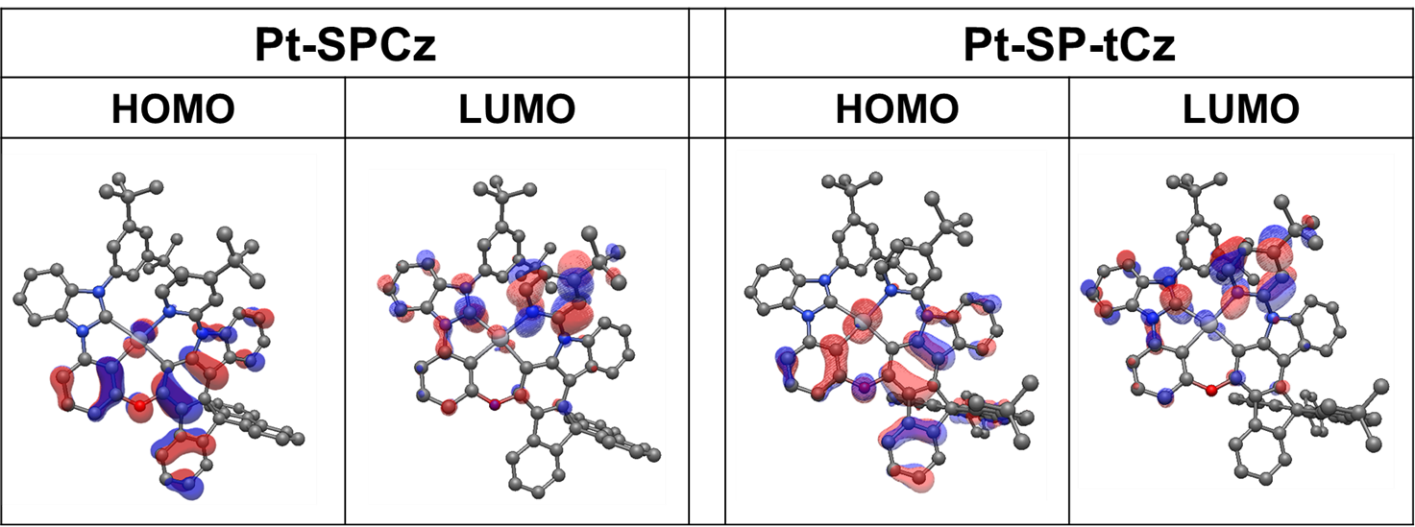
**

**Figure S7. Simulated frontier molecular orbital distributions of Pt-SPCz and Pt-SP-tCz.**

**
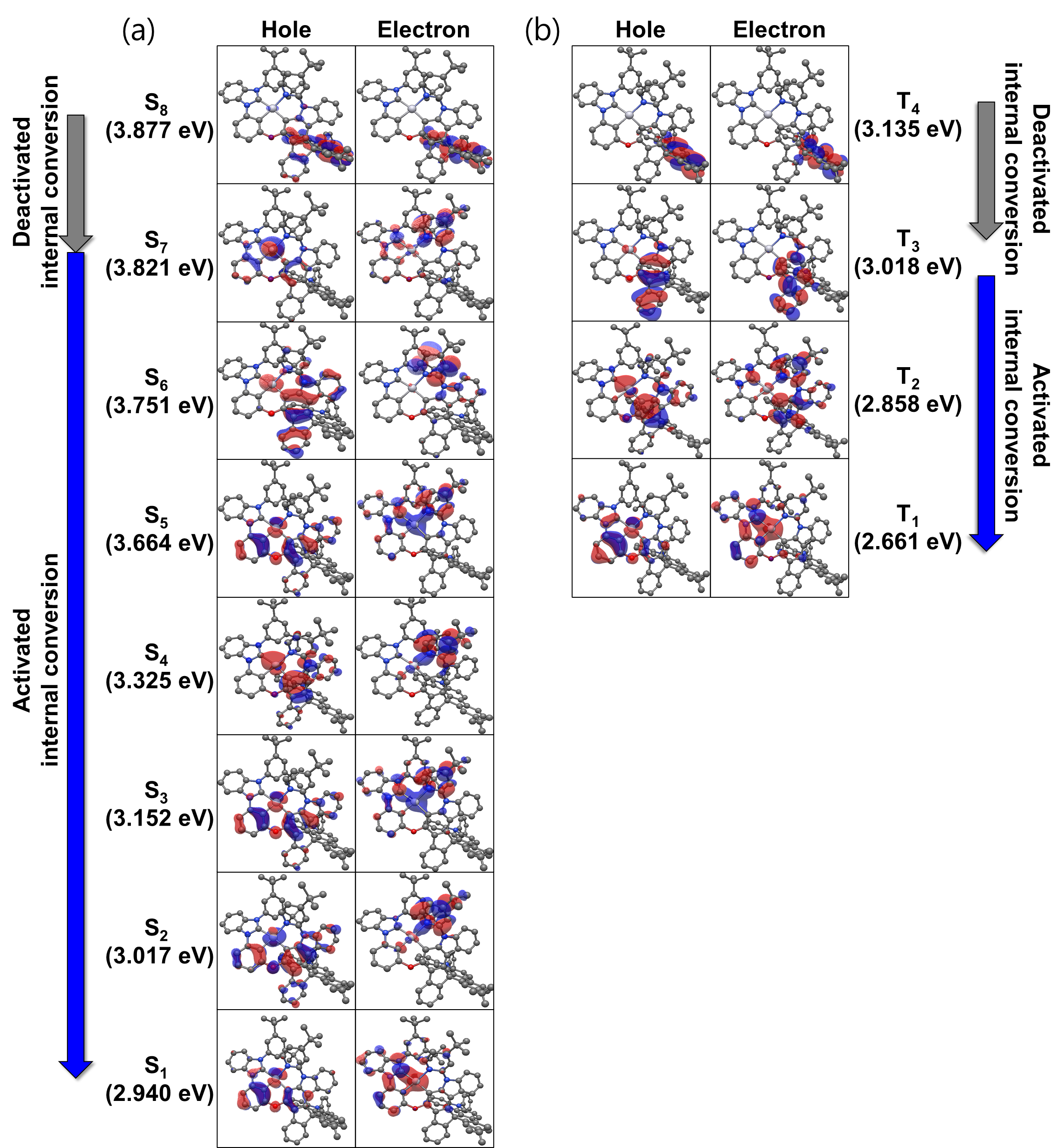
**

**Figure S8. The spatial distribution of hole and electron NTOs in (a) S_n_ (n=1-8) and (b) T_n_ (n=1-4) states and where the corresponding excited state energies and emission parameters.**


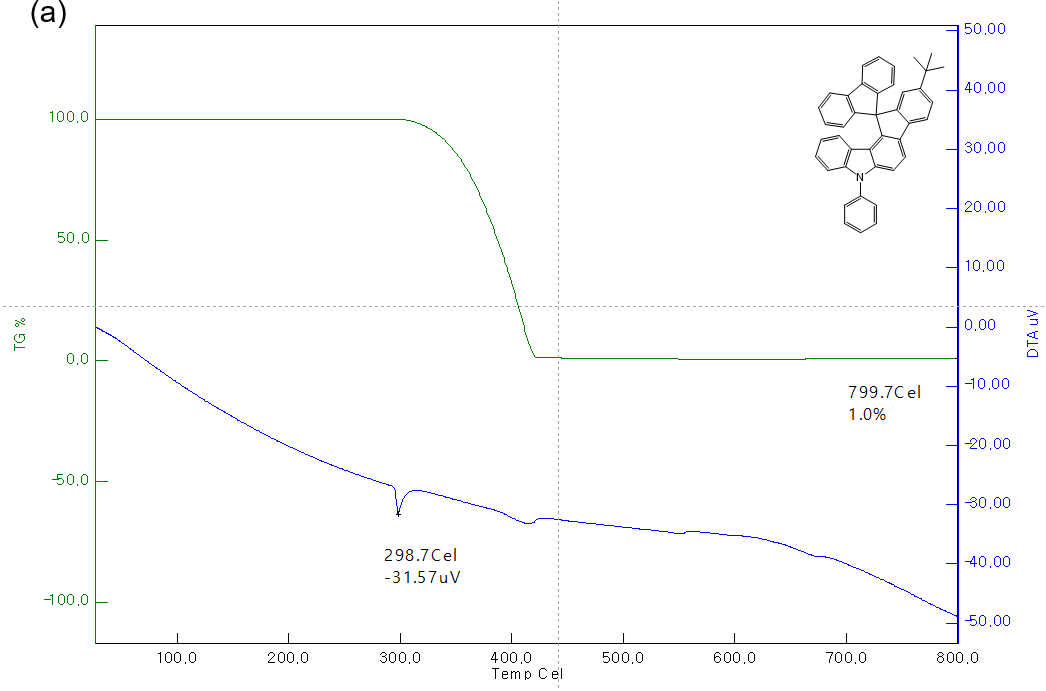

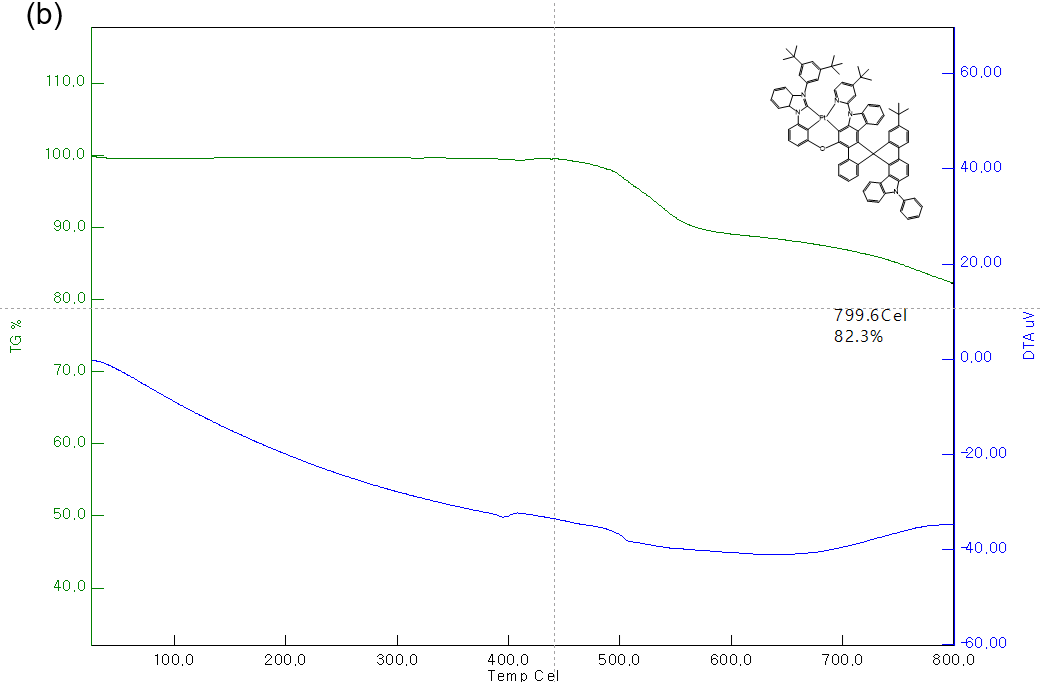


**
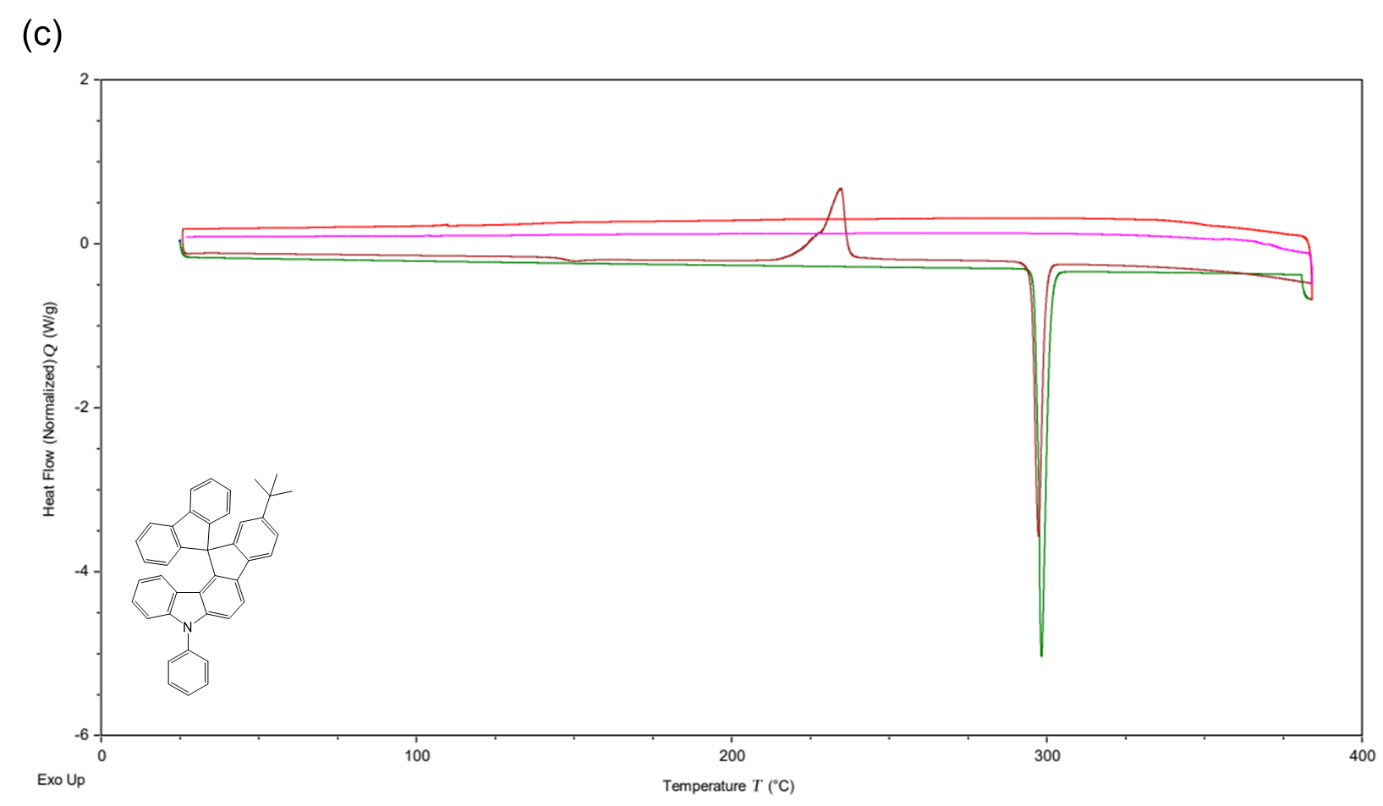
**

**
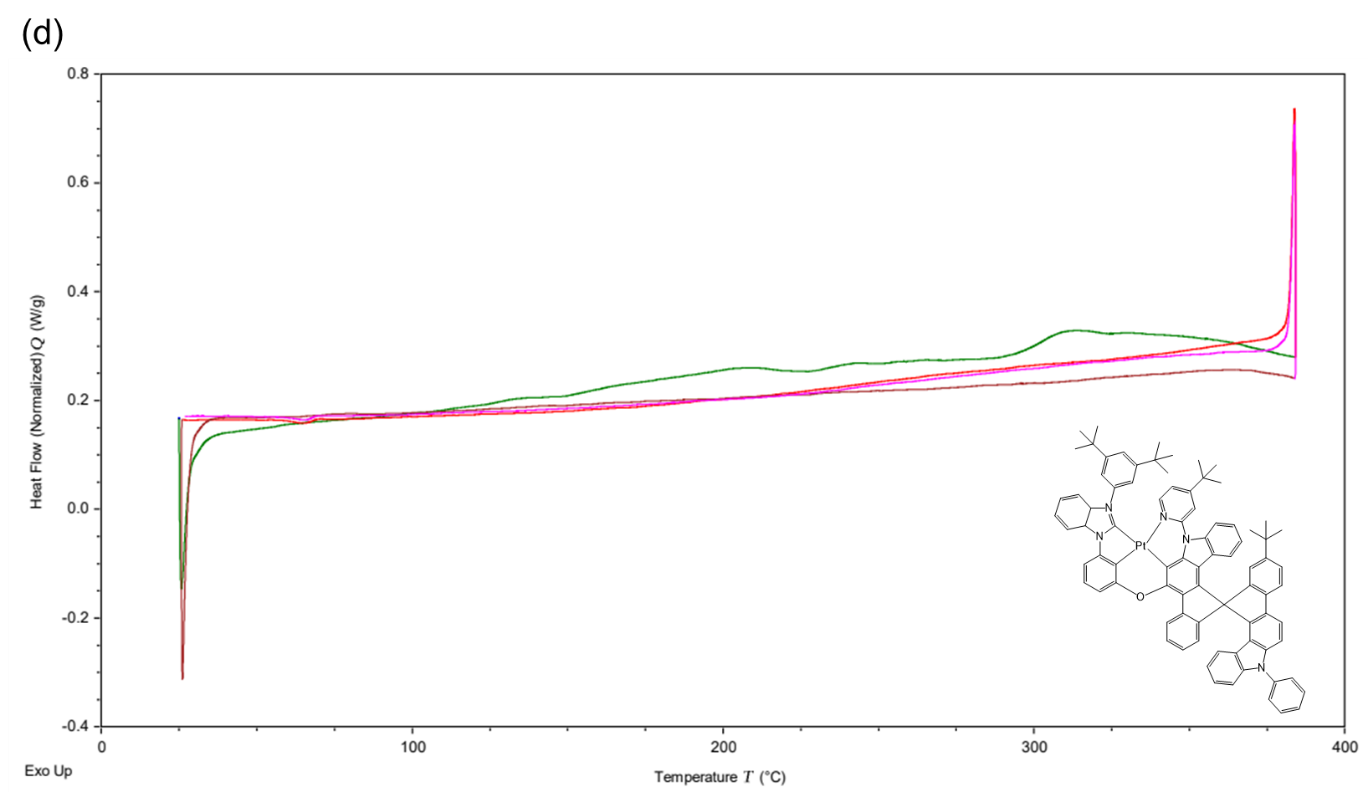
**

**Figure S9. (a,b) TGA and (c-d) DSC of SP-tCz and Pt-SP-tCz. The thermal decomposition temperatures corresponding to 5% weight loss (T_d_) for SP-tCz and Pt-SP-tCz are measured to be 330 ℃, 520 ℃, respectively. The glass transition temperatures (T_g_) of SP-tCz are determined to be 235 ℃. The DSC profile of Pt-SP-tCz show negligible thermal transitions, indicating its thermal stable and amorphous characteristics.**

**
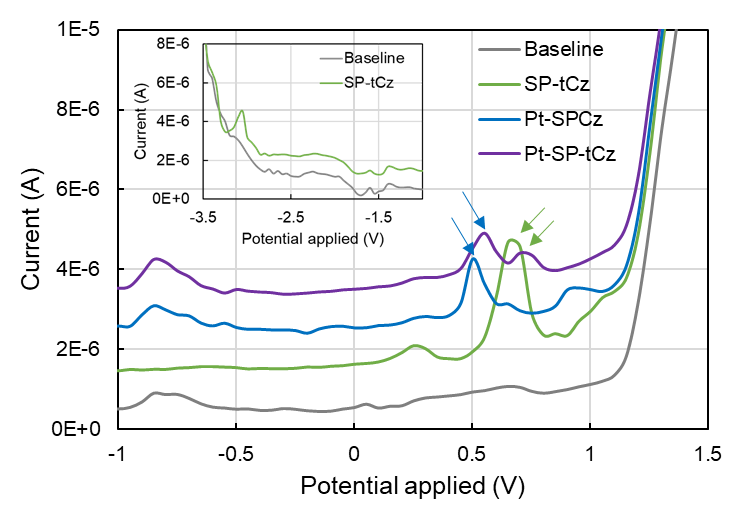
**

**Figure S10. The DPV curves of SP-tCz, Pt-SPCz, and Pt-SP-tCz. The green arrows indicate the peaks associated with the HOMO levels of SP-tCz, while the blue arrows correspond to the HOMO-related peaks of Pt-SPCz. The peaks corresponding to the HOMO level are observed at 0.62 V for SP-tCz, 0.5 V for Pt-SPCz, and 0.53 V for Pt-SP-tCz. The inset figure displays the DPV curve used to determine the LUMO level of SP-tCz, with a distinct peak detected at -3.05 V.**

**
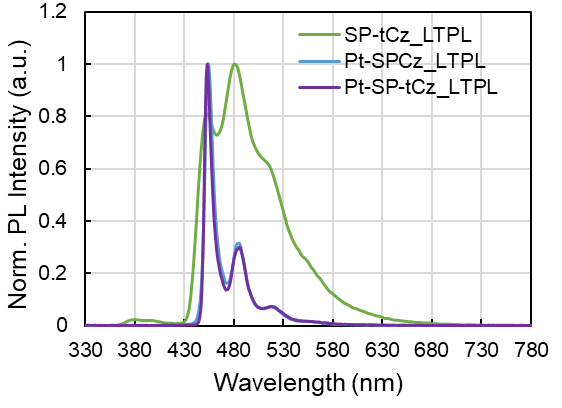
**

**Figure S11. The PL spectra of SP-tCz, Pt-SPCz, Pt-SP-tCz. All samples were prepared in toluene solution a concentration of 2.0 x 10^-5^ M and measured at 77K.**

**
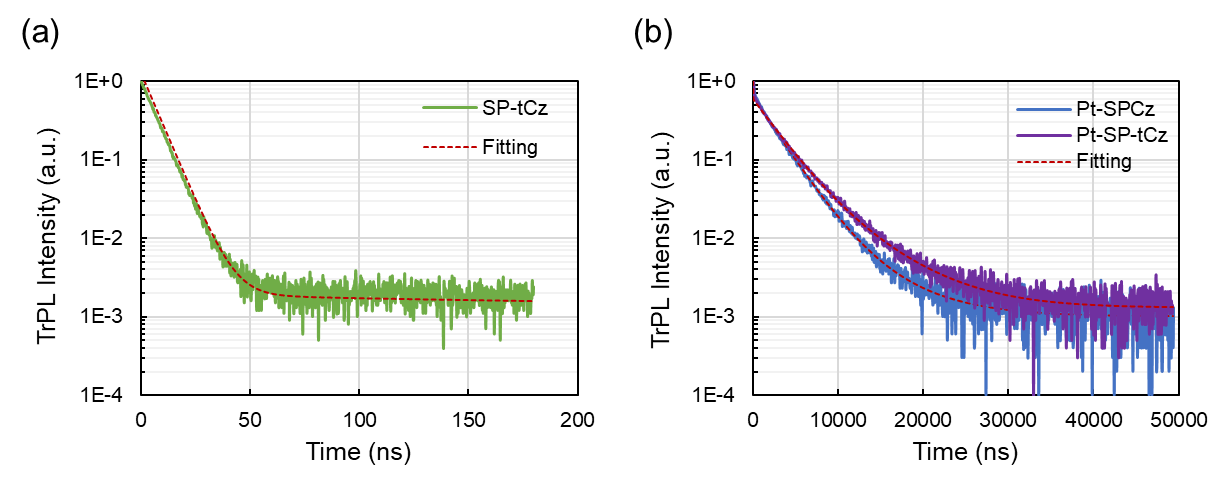
**

**Figure S12. The TrPL decay curves and fitting data of SP-tCz, Pt-SPCz, and Pt-SP-tCz. TrPL was measured for Pt-SPCz and Pt-SP-tCz doped at 10 wt.% into the SP-tCz:SiTrzCz2 mixed host in the film state.**

**Table S1. Physical characteristics of SP-tCz, Pt-SPCz, and Pt-SP-tCz.**

|  | *𝛌_abs_***^a)^**  **[nm]** | ***^𝛌^_PL_*^b)^ [nm]** | **FWHM^c)^ [nm]** | **Φ_PL_^d)^**  **[%]** | **E_g_^e)^ [eV]** | **HOMO^f)/g)^ [eV[** | **LUMO^f)/g)^ [eV]** | **T_g_^h)^/T_d_^i)^**  **[℃]** | **S_1_^j)^/T_1_^k)^**  **[eV]** | **τ^l)^**  **[μs]** |
| --- | --- | --- | --- | --- | --- | --- | --- | --- | --- | --- |
| **SP-tCz** | 320 | 378 | 37 | 45.3 | 3.25 | -5.45/ | -1.75/ | 235/330 | 3.38/2.87 | 0.00665 |
| **Pt-SPCz** | 355/400 | 459 | 17 | 95.1 | 2.88 | -5.30/-4.91 | -2.43/-1.47 |  | /2.77 | 2.57 |
| **Pt-SP-tCz** | 355/400 | 459 | 16 | 94.4 | 2.88 | -5.33/-4.93 | -2.46/-1.51 | /520 | /2.77 | 2.77 |

a) Absorption peak wavelength. b) PL peak wavelength. c) Full width at half maximum of PL spectra. d) Absolute PL quantum yield. e) Optical band gap estimated from the onset of the absorption spectra. f) HOMO or LUMO level extracted from DPV measurements. g) HOMO or LUMO level extracted from DFT simulation. h) Glass transition temperature. i) thermal decomposition temperature at which 5% weight loss occurs. j) Singlet energy level determined from the onset of the PL spectrum measured at RT. k) Triplet energy level determined from the onset of the PL spectrum at 77K. l) Decay time.

**
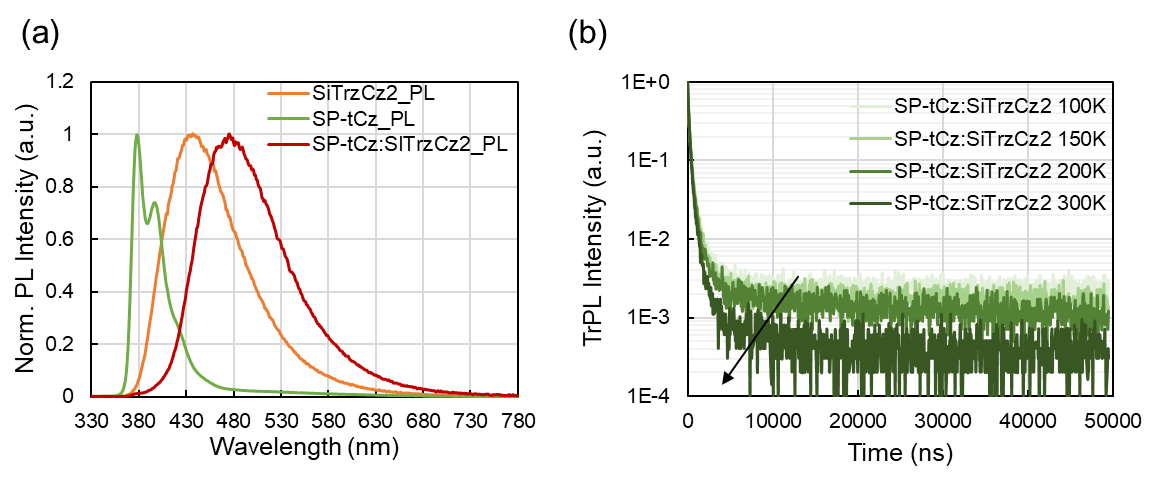
**

**Figure S13. (a) The PL spectra of the SiTrzCz2, SP-tCz, and the SP-tCz:SiTrzCz2 and (b) Temperature-dependent TrPL decay curves measured from 100 K to 300 K for SP-tCz:SiTrzCz2. All samples were measured in thin-film form. The PL spectrum of the SP-tCz:SiTrzCz2 mixed film peak at 479 nm.**

**
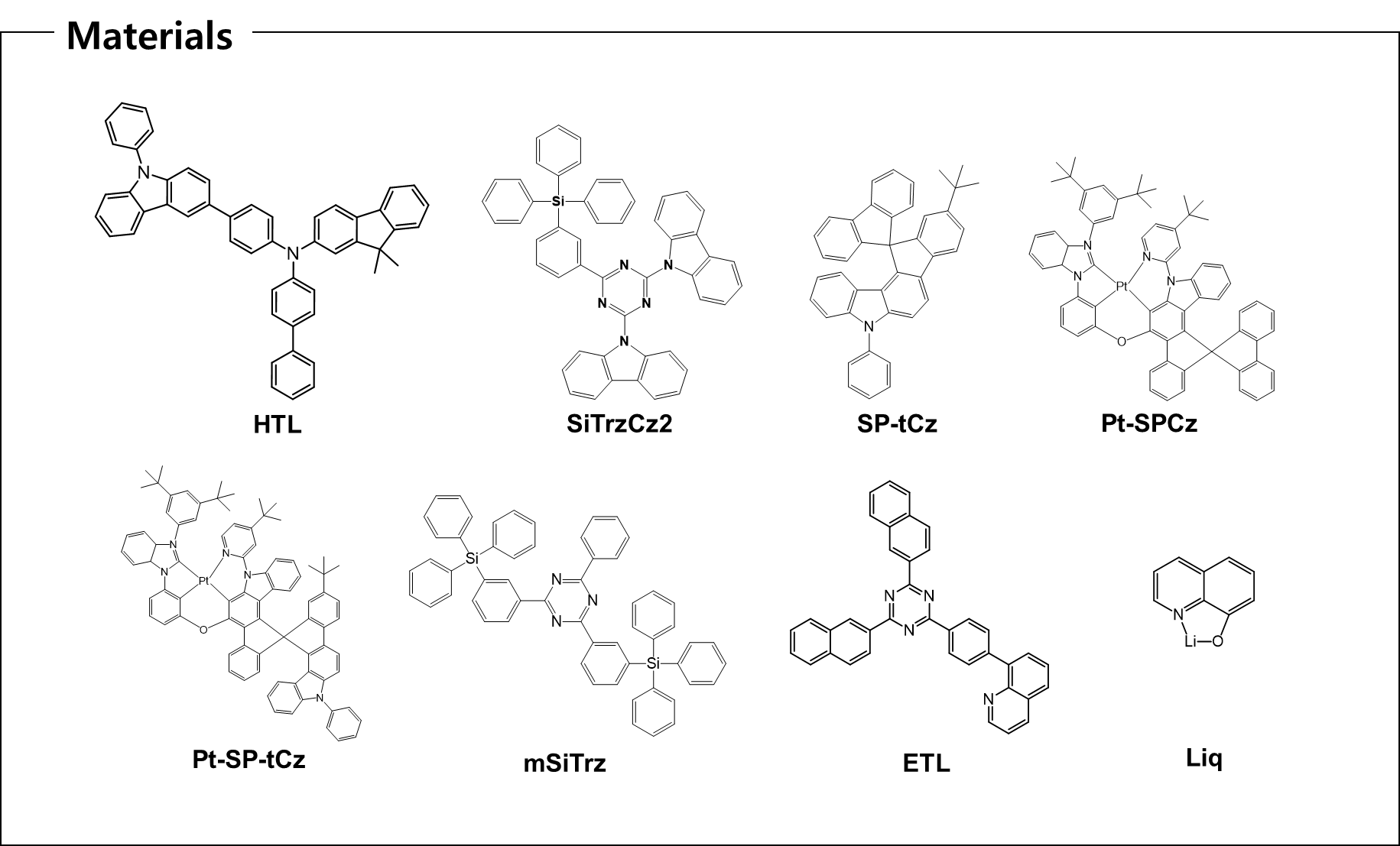
**

**Figure S14. The molecular structures of the materials employed in the fabricated OLED devices.**

N-([1,1′-Biphenyl]-4-yl)-9,9-dimethyl-N-(4-(9-phenyl-9H-carbazol-3-yl)phenyl)-9H-fluoren-2-amine(BCFN) was employed as the HTL and BCFN doped with 3 wt.% of a *p*-dopant was utilized as the HIL. The EBL utilized SP-tCz, which is identical to the p-type host material in the EML, while 9,9′-(6-(3-(triphenylsilyl)phenyl)-1,3,5-triazine-2,4-diyl)bis(9H-carbazole) (SiTrzCz2) was used as the n-type host. As phosphorescent dopants, Pt-SPCz, or Pt-SP-tCz were adopted. For the HBL, 2-Phenyl-4,6-bis(3-(triphenylsilyl)phenyl)-1,3,5-triazine (mSiTrz) was employed. The ETL consisted of a 0.5:0.5 mixture of mSiTrz and 8-Hydroxyquinolinolato-lithium (Liq), with Liq also functioning as the EIL.

**
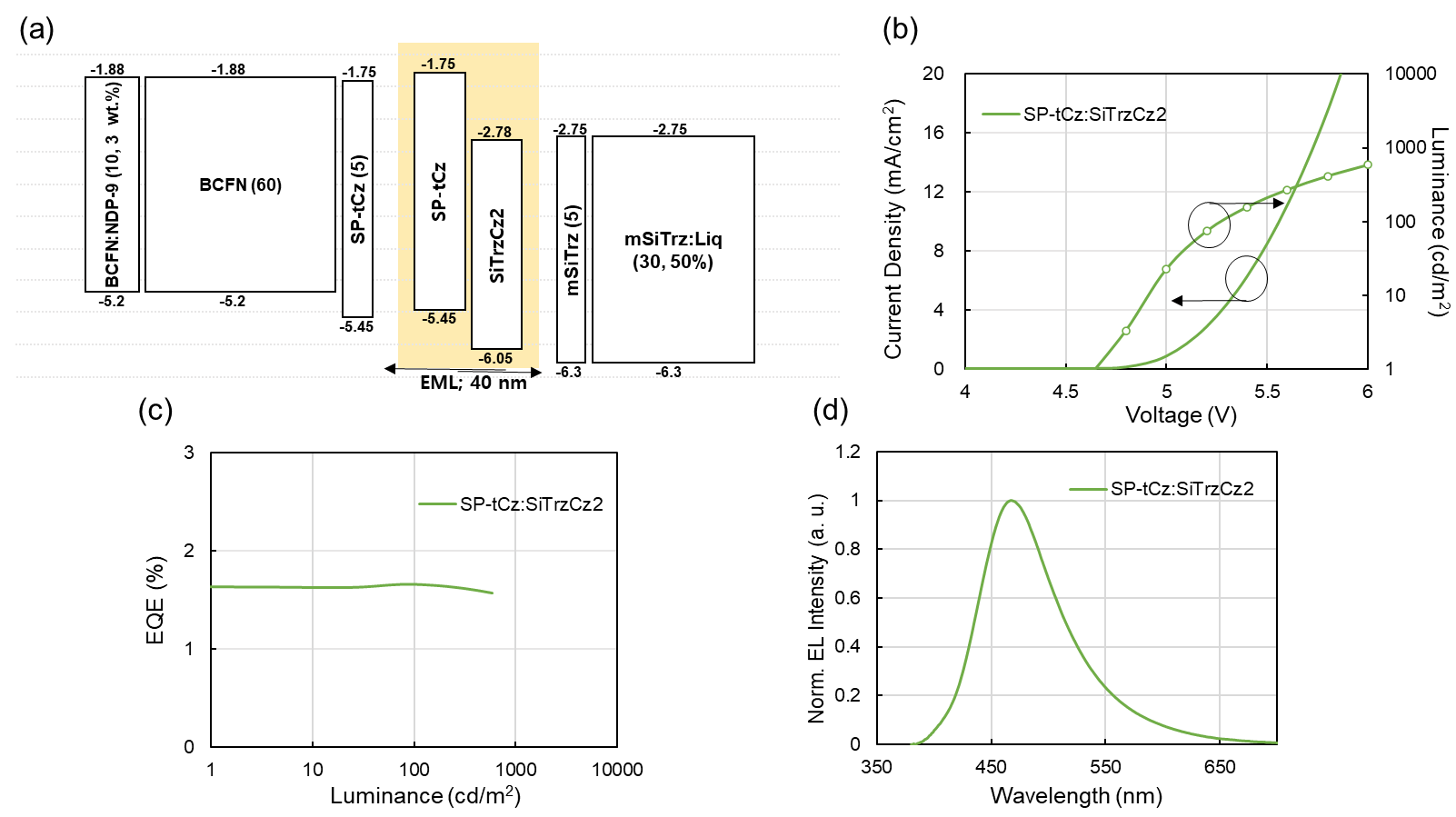
**

**Figure S15. (a) Device structure, (b) *J-V-L* characteristics, (c) EQE curve, and (d) EL spectrum of the fabricated non-doped OLED. The mixing ratio of p-type and n-type hosts was set to 0.55:0.45.**

**Table S2. EL performances of non-doped device.**

| **Device** | **Voltage ^a)^**  **[V]** | **CE ^a)^**  **[cd/A]** | **PE ^a)^**  **[lm/W]** | **Luminance ^a)^**  **[cd/m^2^]** | **EQE ^a)/b)^**  **[%]** | **FWHM ^c)^**  **[nm]** | **CIE_X_^a)^** | **CIE_Y_^a)^** | **λ_max_^a)^**  **[nm]** |
| --- | --- | --- | --- | --- | --- | --- | --- | --- | --- |
| **SP-tCz:SiTrzCz2** | 5.56 | 2.46 | 1.39 | 245.46 | 1.6/1.7 | 80 | 0.177 | 0.230 | 467 |

1. measured at 10 mA/cm^2^_,_ b) max value,c) at 1000 cd/m^2^

**
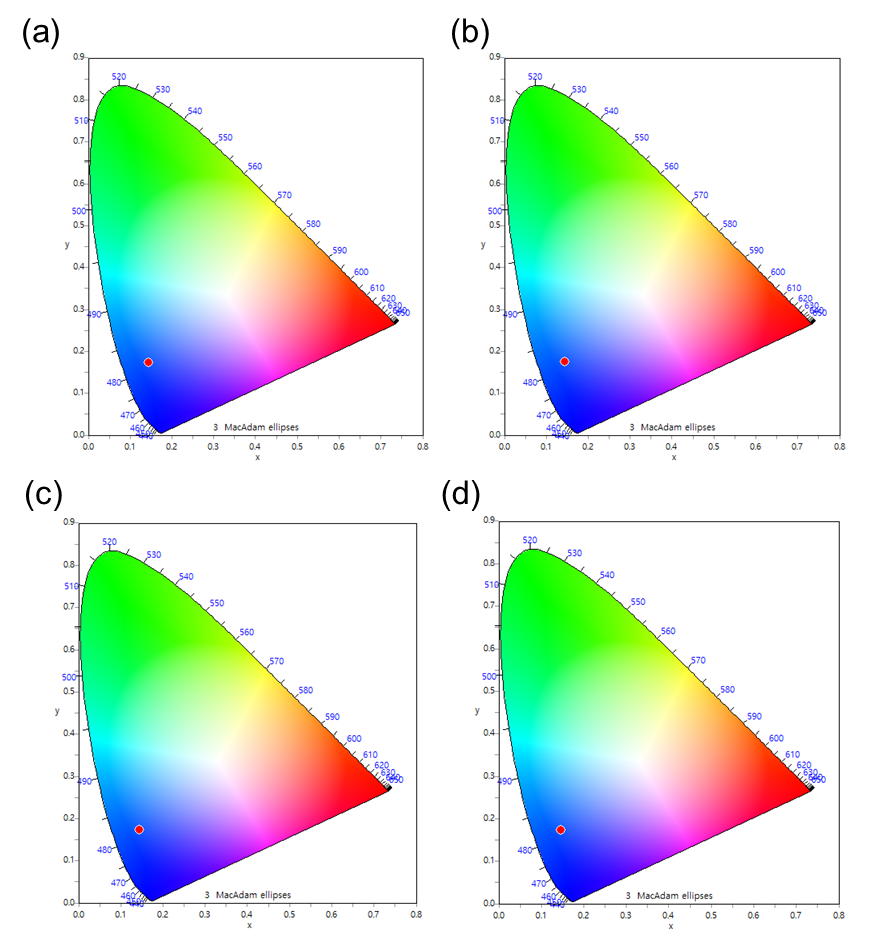
**

**Figure S16. CIE diagrams of the devices based on (a) A_SP-tCz:SiTrzCz2 (1:8), (b) A_SP-tCz:SiTrzCz2 (3:6), (c) A_SP-tCz:SiTrzCz2 (5:4), and (d) A_SP-tCz:SiTrzCz2 (6:3).**

**
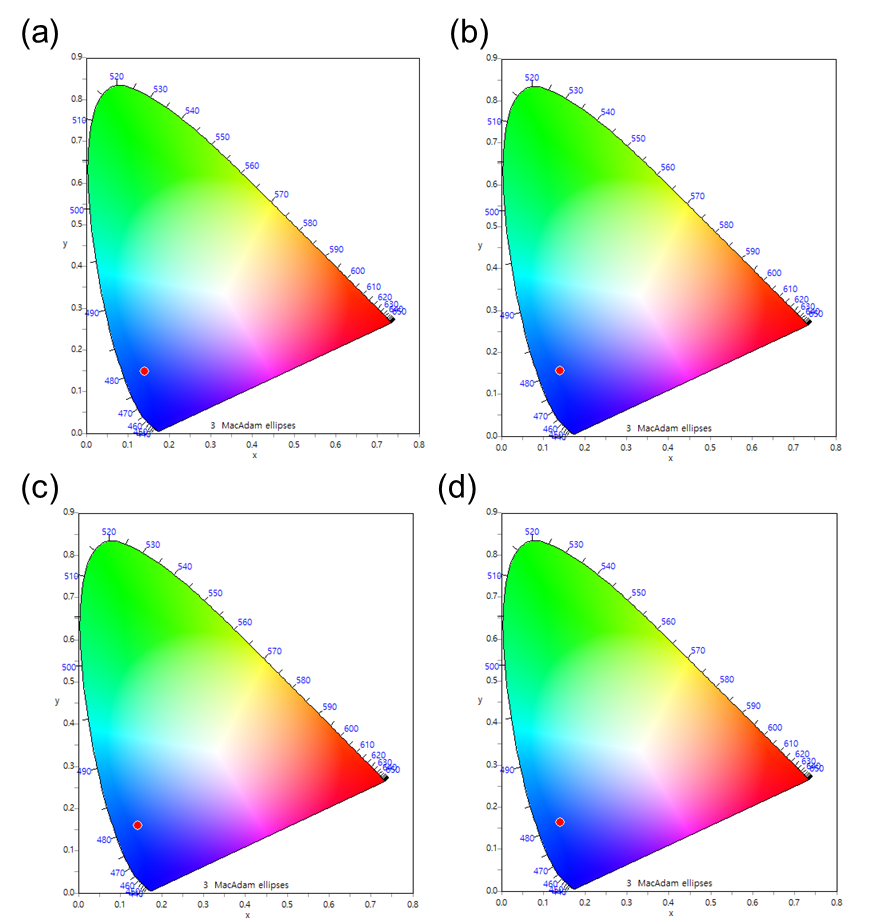
**

**Figure S17. CIE diagrams of the devices based on (a) B_Pt-SP-tCz 5 wt.%, (b) B_Pt-SP-tCz 10 wt.%, (c) B_Pt-SP-tCz 15 wt.%, and (d) Pt-SPCz (2-component).**

**
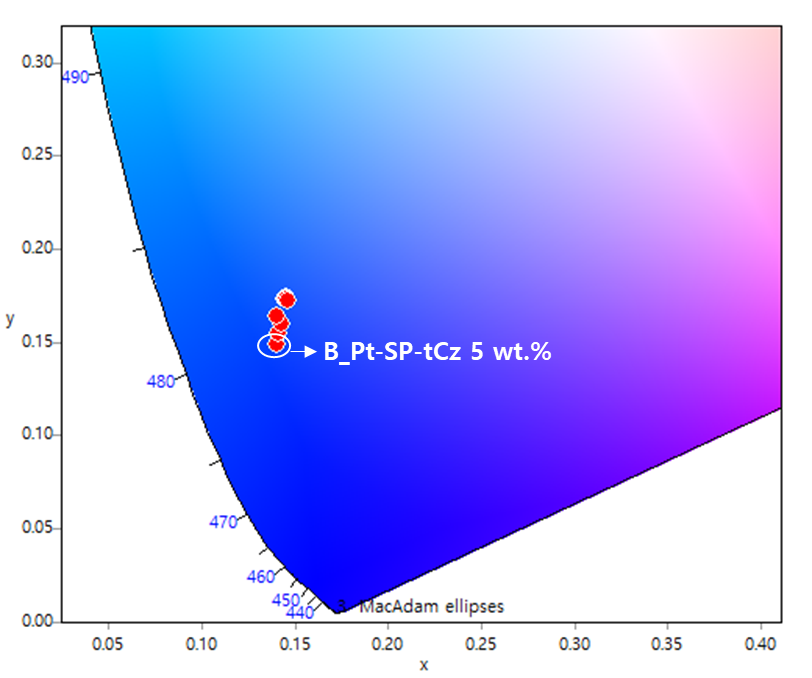
**

**Figure S18. CIE diagram summarizing the color coordinates of the PhOLEDs.**

**
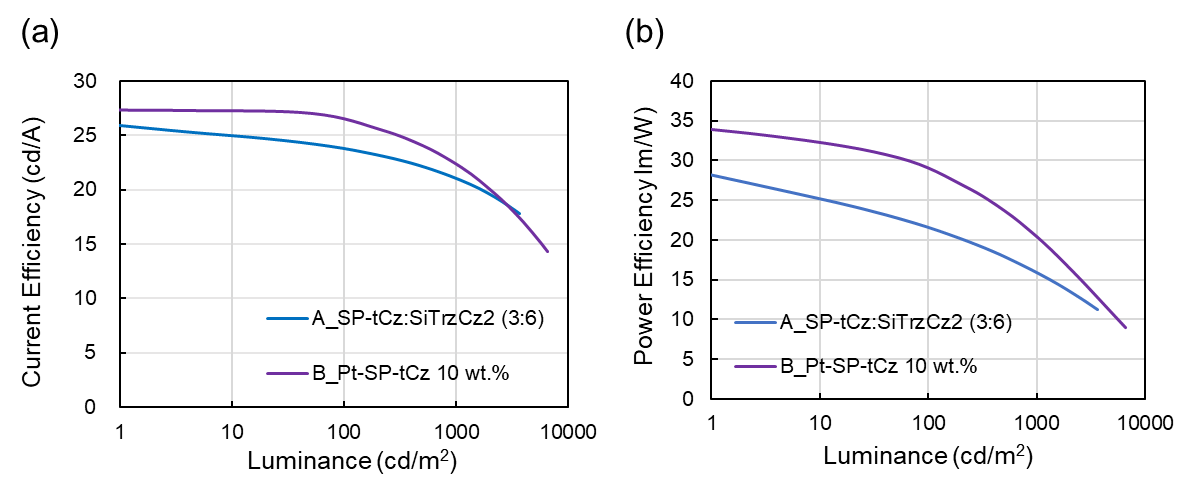
**

**Figure S19. (a) Current efficiency curves and (b) power efficiency curves of the A_SP-tCz:SiTrzCz2 (3:6), and B_Pt-SP-tCz 10 wt.% devices.**

**
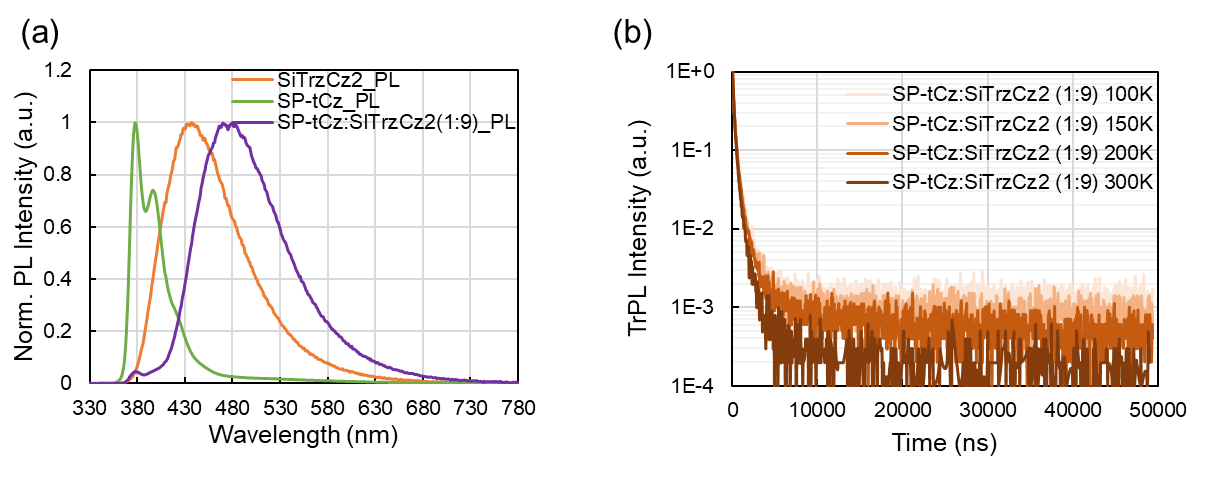
**

**Figure S20. (a) PL spectra of SiTrzCz2, SP-tCz, and the SP-tCz:SiTrzCz2 (0.1:0.9), (b) Temperature-dependent TrPL decay curves measured from 100 K to 300 K for SP-tCz:SiTrzCz2 (0.1:0.9). All samples were measured in thin-film form.**

**
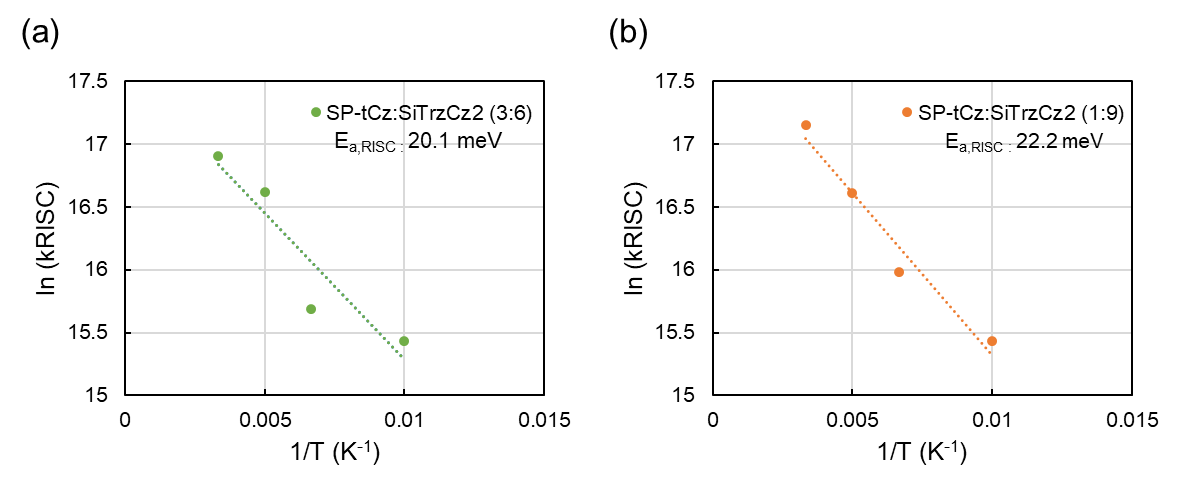
**

**Figure S21. (a) RISC activation energy (E_a,RISC_) extracted from temperature-dependent TrPL measurements of the SP-tCz:SiTrzCz2 (3:6) film. (b) E_a,RISC_ extracted from temperature-dependent TrPL measurements of the SP-tCz:SiTrzCz2 (1:9) film. Ea,risc values were calculated using the equation** $\boldsymbol{k}_{\boldsymbol{RISC}}\boldsymbol{=A\times}\boldsymbol{e}^{\boldsymbol{-}\frac{\boldsymbol{E}_{\boldsymbol{a}}}{\boldsymbol{K}_{\boldsymbol{bT}}}}$**_._**

**
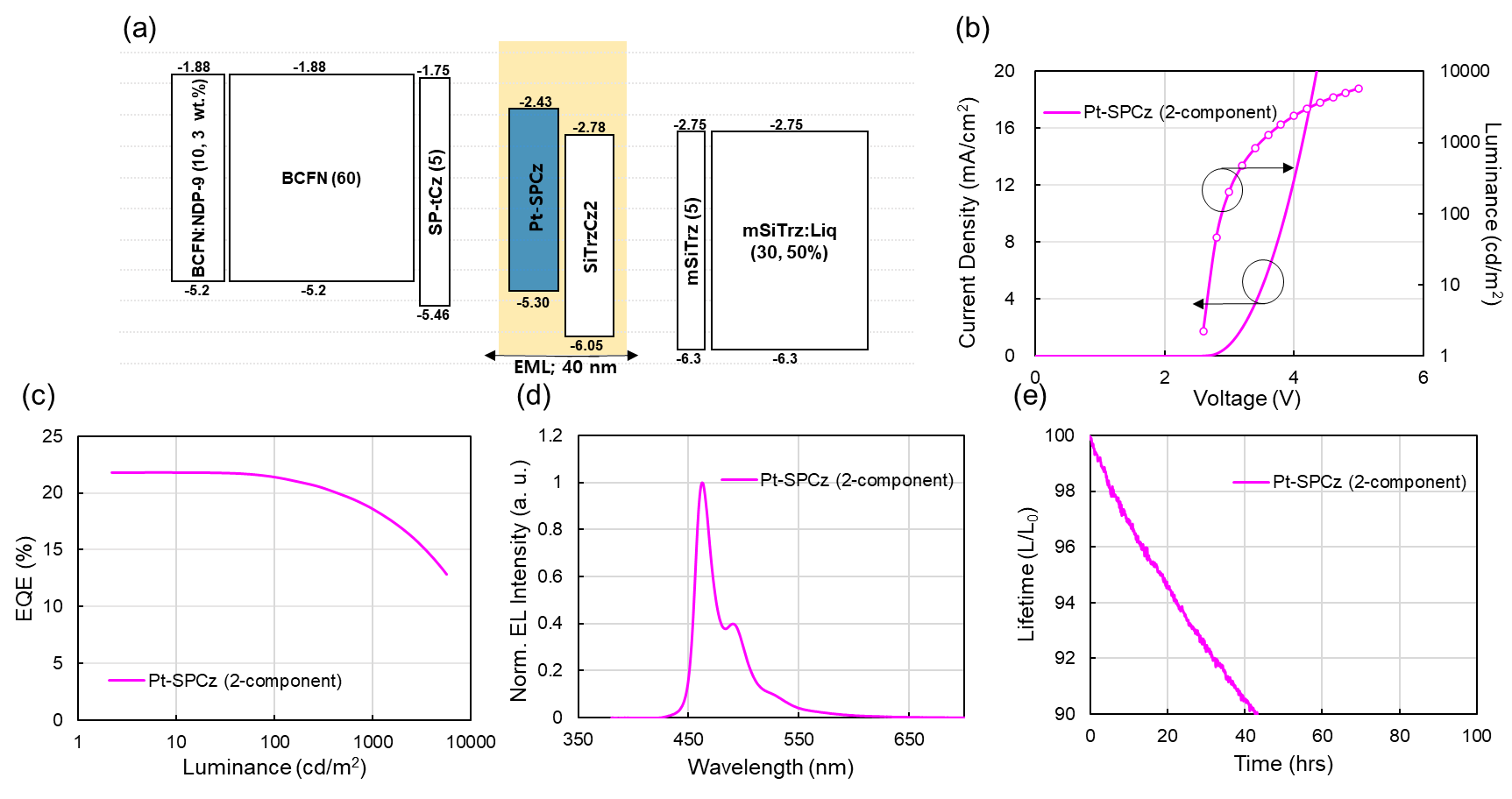
**

**Figure S22. (a) Device structure, (b) *J-V-L* characteristics, (c) EQE curves, (d) EL spectra, and (e) Lifetime of the fabricated 2-componenet EML PhOLEDs. The EMLs of C-series devices are composed of the n-type host SiTrzCz2 and phosphorescent emitter (SiTrzCz2:emitter, 0.9:0.1). Lifetime was assessed based on LT_90_ values (The time required for luminance to decay to 90% of the initial value, measured at 500 nits).**

**Table S3. EL performances of PhOLEDs**

| **Device** | **Voltage ^a)^**  **[V]** | **CE ^a)^**  **[cd/A]** | **PE ^a)^**  **[lm/W]** | **Luminance ^a)^**  **[cd/m^2^]** | **EQE ^a)/b)^**  **[%]** | **FWHM^c)^**  **[nm]** | **CIE_X_^a)^** | **CIE_Y_^a)^** | **λ_max_^a)^**  **[nm]** | **LT_90_^d)^**  **[hrs.]** |
| --- | --- | --- | --- | --- | --- | --- | --- | --- | --- | --- |
| **A_SP-tCz:SiTrzCz2 (1:8)** | 4.32 | 16.40 | 11.94 | 1634.50 | 13.4/19.7 | 21 | 0.144 | 0.173 | 463 | 90 |
| **A_SP-tCz:SiTrzCz2 (3:6)** | 4.52 | 19.63 | 13.66 | 1956.70 | 15.9/21.9 | 21 | 0.145 | 0.174 | 463 | 84 |
| **A_SP-tCz:SiTrzCz2 (5:4)** | 4.61 | 17.10 | 11.66 | 1708.65 | 14.2/17.9 | 21 | 0.145 | 0.173 | 463 | 24 |
| **A_SP-tCz:SiTrzCz2 (6:3)** | 4.67 | 14.45 | 9.74 | 1435.77 | 10.1/16.1 | 21 | 0.146 | 0.172 | 463 | 15 |
| **B_Pt-SP-tCz 5 wt.%** | 3.88 | 16.52 | 13.41 | 1643.23 | 14.6/21.1 | 18 | 0.140 | 0.149 | 461 | 10 |
| **B_Pt-SP-tCz 10 wt.%** | 3.83 | 20.07 | 16.48 | 2001.68 | 17.2/23.6 | 19 | 0.141 | 0.155 | 461 | 82 |
| **B_Pt-SP-tCz 15 wt.%** | 3.62 | 19.73 | 17.12 | 1967.73 | 16.3/21.2 | 19 | 0.143 | 0.160 | 462 | 31 |
| **Pt-SPCz (2-component)** | 3.86 | 19.61 | 15.99 | 1953.87 | 16.4/21.8 | 20 | 0.140 | 0.164 | 462 | 44 |

a) measured at 10 mA/cm^2^_,_ b) max value, c) at 1000 cd/m^2^, d) at 500 cd/m^2^

**
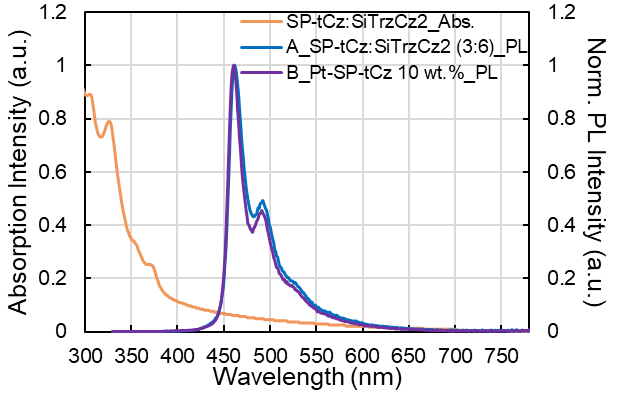
**

**Figure S23. The UV-vis absorption spectrum of SP-tCz:SiTrzCz2 and the normalized PL spectra of films with the same EML composition as the A_SP-tCz:SiTrzCz2_(3:6) and B_Pt-SP-tCz_10 wt.% devices. The PL spectra were obtained by exciting the samples at 310 nm. All samples were measured in thin-film form.**

**
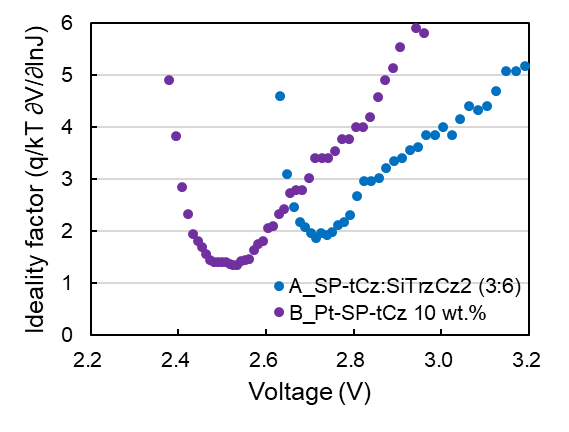
**

**Figure S24. Ideality factors extracted from A_SP-tCz:SiTrzCz2 (3:6) and B_Pt-SP-tCz 10 wt.% devices.**

**Calculation of Polaron and Exciton Dynamics**

The prompt and delayed TrPL profiles were fitted via double exponential decay equations^[7]^ as follows:

$I_{P}\left( t \right)$ = A_P1_exp($-k_{P1}t)$ + A_P2_exp($-k_{P2}t)$ + $I\left( 0 \right)$

$I_{D}\left( t \right)$ = A_D1_exp($-k_{D1}t)$ + A­_D2_exp($-k_{D2}t)$

where, $I_{P}\left( t \right)$, $I_{D}\left( t \right)$, A_P1,_ A_P2,_ A_D1,_ and A_D2_ represents prompt PL intensity, delayed PL intensity, prompt amplitudes and delayed amplitudes for the respective decay components. $k_{P1}$, $k_{P2}$, $k_{D1}$, and $k_{D2}$ represents prompt exciton decay rate coefficients and delayed exciton decay rate coefficients for the respective decay components. The average rate coefficients ($k_{P}$ and $k_{D}$) were determined using intensity-weighted decay time as follows:

$\tau=\frac{\sum_{\dot{i}=1}^{n} \tau_{i}^{2}A_{i}}{\sum_{\dot{i}=1}^{n} \tau_{i}A_{i}}$

where, τ represent intensity-weighted decay time. From prompt and delayed exciton decay rate coefficients ($k_{P}= \frac{1}{\tau_{P}}$ and $k_{D}= \frac{1}{\tau_{D}}$) with photoluminescence quantum yield values, monomolecular rate coefficients ($k_{r,H}$,$k_{FRET,H}$, $k_{r,D}$, and $k_{nr,D}$) were calculated.^[8]^ $k_{r,H}$,$k_{FRET,H}$, $k_{r,D}$, and $k_{nr,D}$ represents radiative singlet rate coefficient of host, FRET rate coefficient from host to dopant, radiative and non-radiative triplet rate coefficients of dopant.

The trEL profiles of exciplex, 3 components, and 2 components devices were measured at current density of 5 mA cm^-2^ with voltage pulse of 500 $\mu$s and pulse-off of 500 $\mu$s. The trEL fitting was performed using following equations.

$\frac{\left. \partial n(t \right)}{\partial t}=\frac{j}{ed}-\gamma\left. n(t \right)^{2}$

$\frac{\left. ⅆS_{H}(t \right)}{ⅆt}=-\left( k_{r,H}+k_{ISC,H}+k_{FRET,H} \right)S_{H}\left( t \right)+k_{RISC,H}T_{H}\left( t \right)+\frac{1}{8}k_{TT,H}{T_{H}\left( t \right)}^{2}+\frac{1}{4}\gamma\left. n(t \right)^{2}$

$\frac{\left. ⅆT_{H}(t \right)}{ⅆt}=k_{ISC,H}S_{H}\left( t \right)-(k_{RISC,H}+k_{nr,H})T_{H}\left( t \right)-k_{TP,H}T_{H}\left( t \right)n(t)-\frac{5}{8}k_{TT,H}{T_{H}\left( t \right)}^{2}+\frac{3}{4}\gamma\left. n(t \right)^{2}$

$\frac{\left. ⅆS_{D}(t \right)}{ⅆt}=k_{FRET,H}S_{H}\left( t \right)-k_{ISC,D}S_{D}\left( t \right)$

$\frac{\left. ⅆT_{D}(t \right)}{ⅆt}=k_{ISC,D}S_{D}\left( t \right)-(k_{r,D}+k_{nr,D})T_{D}\left( t \right)-\frac{1}{2}k_{TT,D}{T_{D}\left( t \right)}^{2}$

Here, $\left. n(t \right)$, $\left. S_{H}(t \right)$, $\left. T_{H}(t \right)$, $\left. S_{D}(t \right)$, and $\left. T_{D}(t \right)$ are polaron, host singlet, host triplet, dopant singlet, and dopant triplet density. $k_{ISC,H}$, $k_{RISC,H}$, $k_{nr,H}$, $k_{TT,H}$, $k_{TP,H}$, and $\gamma$ are rate coefficients of intersystem crossing, reverse-intersystem crossing, non-radiative singlet, triplet-triplet annihilation, triplet-polaron annihilation, and polaron recombination in the host. $k_{ISC,D}$ and $k_{TT,D}$ are rate coefficients of intersystem crossing and triplet-triplet annihilation in the dopant. Due to the large number of fitting parameters in the coupled equations, we first determined the first-order reaction rate constants of the host and dopant by fitting the transient PL profiles of the thin films. Subsequently, we extracted the remaining rate constants and the recombination coefficient by fitting the transient EL profiles. A least-squares method was employed as the fitting algorithm. In this work, we assumed that the charge balance factor was unity and that FRET was the dominant energy transfer mechanism rather than Dexter energy transfer. The recombination process was defined by numerical fitting rather than the Langevin recombination rule for a suitable approach.^[9]^

**
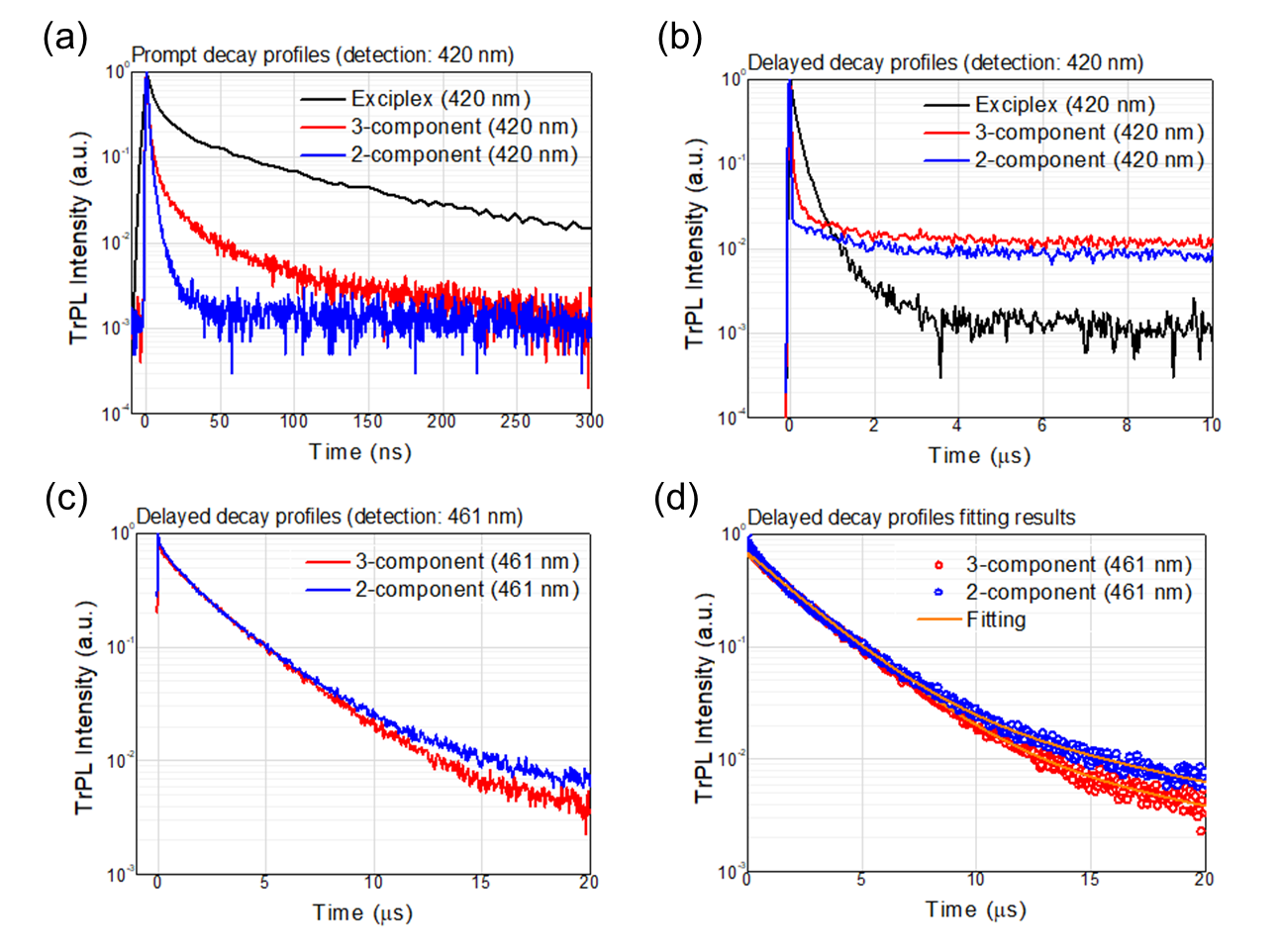
**

**Figure S25. TrPL fitting results. (a) Prompt and (b) delayed TrPL of exciplex, 3 components, and 2 component devices. (c) Delayed TrPL of 3 components and 2 components devices. (d) Fitting result of delayed TrPL profiles of (c).**

**Table S4. Summarized TrPL fitting parameters. The rate coefficients were calculated at the wavelength of 420 nm.**

|  | τ_PF_^(a)^  (ns) | $k_{PF}$^(b)^  ($\times$10^7^ s^-1^) | τ_DF_^(c)^  ($\mu$s) | $k_{DF}$^(d)^  ($\times$10^5^ s^-1^) | $\Phi$  (%) | $\Phi\mathrm{PF}$^(e)^  (%) | $\Phi\mathrm{DF}$^(f)^  (%) | $k_{r,H}$^(g)^  ($\times$10^6^ s^-1^) |
| --- | --- | --- | --- | --- | --- | --- | --- | --- |
| Exciplex | 67.1 | 1.49 | 1.02 | 9.80 | 0.36 | 0.31 | 0.05 | 4.61 |

1. Prompt fluorescence time. (b) Prompt fluorescence rate constant. (c) Delayed fluorescence time. (d) Delayed fluorescence rate constant. (e) Prompt and (f) delayed PLQY measured in exciplex host film. (g) Radiative singlet rate coefficient of host.

**Table S5. Summarized TrPL fitting parameter of 3 components and 2 component films. The rate coefficients were calculated at the wavelength of 461 nm.**

|  | $\Phi$  (%) | $k_{r.D}$^(a)^  ($\times$10^5^ s^-1^) | $k_{nr.D}$^(a)^  ($\times$10^4^ s^-1^) |
| --- | --- | --- | --- |
| 3-component | 0.74 | 1.90 | 6.68 |
| 2-component | 0.88 | 2.07 | 2.82 |

(a) The decay coefficient was calculated from TRPL profile detected at the wavelength of 461 nm.

The DET rates for both systems were calculated using the following equation based on the TrPL decay data.

$$k_{DET}=\frac{1}{\tau_{DF(DA)}}-\frac{1}{\tau_{DF\left( D \right)}}+k_{RISC}k_{ISC}.(\tau_{PF\left( DA \right)}-\tau_{PF\left( D \right)})$$

where *τ_DF(DA)_* and *τ_DF(DA)_* are the delayed fluorescent lifetimes of the host with and without dopant, respectively.

**
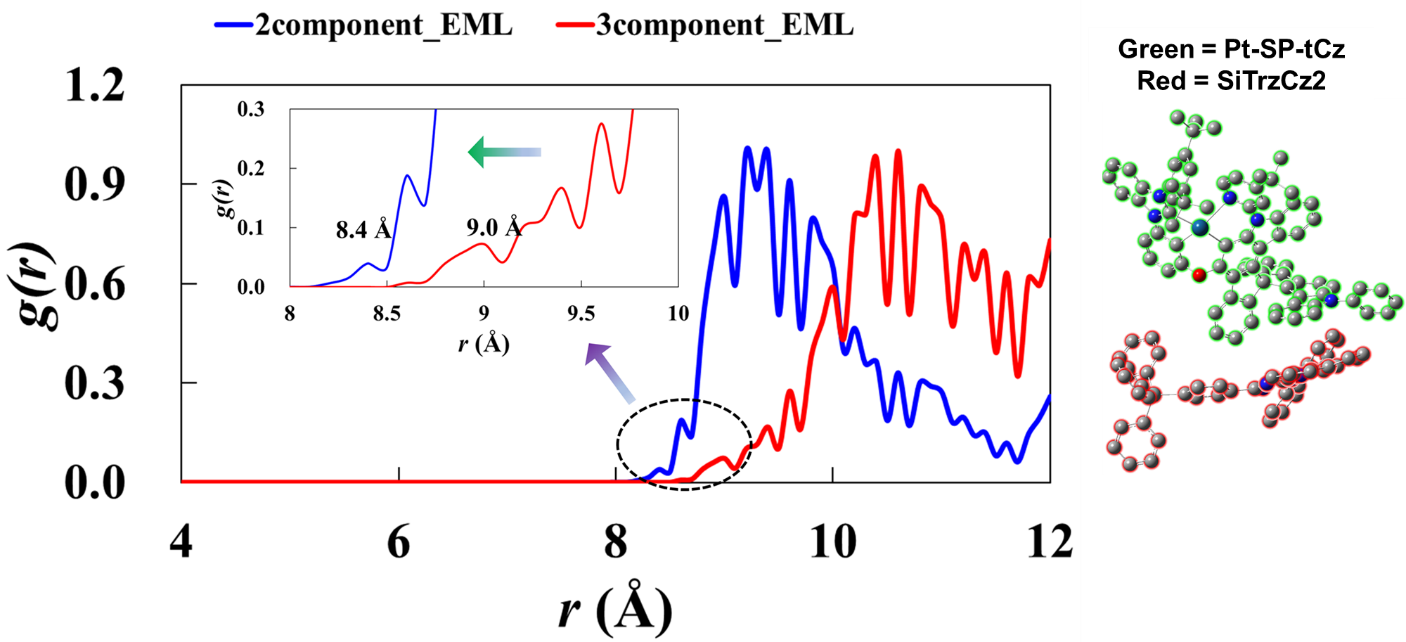
**

**Figure S26. The plotted RDFs of 2-component and 3-component systems.**

**Reference**

[1] M. e. Frisch, G. Trucks, H. B. Schlegel, G. Scuseria, M. Robb, J. Cheeseman, G. Scalmani, V. Barone, G. Petersson, H. Nakatsuji, Gaussian, Inc. Wallingford, CT, 2016.

[2] A. Fetter, J. Walecka, McGraw-Hill, NY, 1971.

[3] a)H. Lee, B. Park, G. R. Han, M. S. Mun, S. Kang, W. P. Hong, H. Y. Oh, T. Kim, *Advanced Materials* **2024**, 36, 2409394; b)K. Cheong, H. Lee, J. Moon, C. H. Ryu, G. W. Kim, J. Y. Kim, I. H. Lee, Y. W. Kim, Y. W. Lee, S. Yu, *Advanced Materials* **2025**, e10070.

[4] F. Neese, *Wiley Interdisciplinary Reviews: Computational Molecular Science* **2025**, 15, e70019.

[5] D. E. McCumber, *Physical Review* **1964**, 136, A954.

[6] K. J. Bowers, E. Chow, H. Xu, R. O. Dror, M. P. Eastwood, B. A. Gregersen, J. L. Klepeis, I. Kolossvary, M. A. Moraes, F. D. Sacerdoti, presented at *Proceedings of the 2006 ACM/IEEE Conference on Supercomputing*, **2006**.

[7] H. S. Kim, S. H. Lee, S. Yoo, C. Adachi, *Nature communications* **2024**, 15, 2267.

[8] a)H. Uoyama, K. Goushi, K. Shizu, H. Nomura, C. Adachi, *Nature* **2012**, 492, 234; b)N. Aizawa, S. Shikita, T. Yasuda, *Chemistry of Materials* **2017**, 29, 7014; c)X. Song, D. Zhang, Y. Zhang, Y. Lu, L. Duan, *Advanced Optical Materials* **2020**, 8, 2000483.

[9] S. Wu, L. Zhang, J. Wang, A. Kumar Gupta, I. D. Samuel, E. Zysman‐Colman, *Angewandte Chemie* **2023**, 135, e202305182.
